# Immune response drives outcomes in prostate cancer: implications for immunotherapy

**DOI:** 10.1002/1878-0261.12887

**Published:** 2020-12-29

**Authors:** Jialin Meng, Yujie Zhou, Xiaofan Lu, Zichen Bian, Yiding Chen, Jun Zhou, Li Zhang, Zongyao Hao, Meng Zhang, Chaozhao Liang

**Affiliations:** ^1^ Department of Urology The First Affiliated Hospital of Anhui Medical University Hefei China; ^2^ Institute of Urology & Anhui Province Key Laboratory of Genitourinary Diseases Anhui Medical University Hefei China; ^3^ Division of Gastroenterology and Hepatology Key Laboratory of Gastroenterology and Hepatology Ministry of Health, Renji Hospital School of Medicine Shanghai Jiao Tong University Shanghai Institute of Digestive Disease China; ^4^ State Key Laboratory of Natural Medicines Research Center of Biostatistics and Computational Pharmacy China Pharmaceutical University Nanjing China; ^5^ Urology Institute of Shenzhen University The Third Affiliated Hospital of Shenzhen University Shenzhen University China

**Keywords:** immune checkpoint blockade therapy, immune molecular subclassification system, immunotherapy, non‐negative matrix factorization, prostate cancer

## Abstract

The heterogeneity of the immune microenvironment leads to different responses in immune checkpoint blockade therapy. We aimed to propose a robust molecular classification system to investigate the relevance of the immune microenvironment subtype and prognosis of prostate cancer patients, as well as the therapeutic response to immune checkpoint blockade therapy. A total of 1,557 prostate cancer patients were enrolled, including 69 real‐world samples from our institute (titled the AHMU‐PC cohort). The non‐negative matrix factorization algorithm was employed to virtually microdissect patients. The immune enrichment was characterized by a high enrichment of T cell‐, B cell‐, NK cell‐, and macrophage‐associated signatures, by which patients were subclassified into nonimmune and immune classes. Subsequently, the immune class was dichotomized into immune‐activated and immune‐suppressed subtypes based on the stromal signature, represented by the activation of WNT/TGF‐β, TGF‐β1, and C‐ECM signatures. Approximately 14.9% to 24.3% of patients belonged to the immune‐activated subtype, which was associated with favorable recurrence‐free survival outcomes. In addition, patients in the immune‐activated subtype were predicted to benefit more from anti‐PD‐1/PD‐L1 therapy. In conclusion, our study identifies a novel immune molecular classifier that is closely related to clinical prognosis and provides novel insights into immunotherapeutic strategies for prostate cancer patients.

## Graphical abstract

This study identifies a novel immune molecular classifier for prostate cancer based on the analysis of 1,557 cases. Patients in the immune activated subgroup were associated with favorable prognosis and better outcomes of anti‐PD‐1/PD‐L1 therapy. Our findings suggest that the immune response drives clinical outcomes in prostate cancer, highlighting important implications for immunotherapy in patients with prostate cancer.

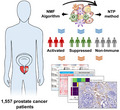

**Abbreviations**
ADT: androgen deprivation therapy
CNAs: copy number alterations
CRPC: castration‐resistant prostate cancer
CYT: cytolytic activity score
DEGs: differentially expressed genes
FDA: the Food and Drug Administration
FDR: false discovery rate
FFPE: formalin‐fixed, paraffin‐embedded samples
ICB: immune checkpoint blockade
IHC: immunohistochemistry
MDS: multidimensional scaling
NMF: non‐negative matrix factorization
NTP: nearest template prediction
OS: overall survival
PD‐1: programmed cell death protein 1
PD‐L1: programmed cell death 1 ligand 1
PD‐L2: programmed cell death 1 ligand 2
TILs: tumor‐infiltrating lymphocytes
TLS: tertiary lymphoid structure
TME: tumor microenvironment

## 1 Introduction

Since prostate cancer is the second most common tumor and ranks as the fifth most common reason of cancer‐related death among males, its substantial worldwide burden has raised public health concerns [1]. The outcomes of low‐intermediate‐risk patients are favorable with the application of minimally invasive ablative therapies, radiation therapy, or radical prostatectomy. However, approximately 26% to 30% of prostate cancer patients will develop to advanced and metastatic disease within five years [2]. Although androgen deprivation therapy (ADT) is available for advanced‐stage patients [3], they still experience unfavorable outcomes due to the rapid progression to castration‐resistant prostate cancer (CRPC), which can cause prostate cancer‐specific death within 2 to 4 years [4]. For CRPC patients who received maximum androgen blockade therapy, the 5‐year overall survival (OS) rate is 25.4%, while for patients who received only androgen suppression by surgery, it is 1.8% [5]. Currently, sipuleucel‐T, abiraterone acetate, enzalutamide, cabazitaxel, radium‐223, and apalutamide treatments are approved by the Food and Drug Administration (FDA) and are available for CRPC patients.

The tumor microenvironment (TME) can also be regarded as the tumor milieu, a composite of blood vessels, immune cells, stromal cells, mesenchymal cells, cytokines, and chemokines [6], and it plays a crucial role in tumorigenesis and tumor progression. Many investigations have explored the role of the TME in tumor progression and prognostic prediction. In our previous study, we found that polarizated M2 macrophage can be a risk factor for prostate cancer patients [7]. Zhao *et al*. [8] demonstrated the association between high expression levels of programmed cell death 1 ligand 2 (PD‐L2) and poor outcomes in prostate cancer patients, as well as its link with postoperative radiation therapy. Rodrigues *et al*. [9] also illustrated the positive association between defects in mismatch repair pathways and the overactivation of several immune checkpoints. Sipuleucel‐T is the first FDA‐approved immunotherapy for prostate cancer patients, the recombinant fusion prostatic acid phosphatase (PAP) can activate antigen‐presenting cells (APCs) and shift the immunosuppressive milieu of tumors [10]. Anti‐programmed cell death protein 1 (PD‐1) and anti‐programmed cell death 1 ligand 1 (PD‐L1) therapy is another potential immunotherapy option for prostate cancer patients and has been confirmed to offer benefits to patients with melanoma, nonsmall cell lung cancer, breast cancer, and urothelial carcinoma. However, only some patients respond to these immune checkpoint blockade (ICB) treatments, and the molecular features of the TME are tightly linked to patients’ response to chemoradiotherapy and ICB [11]. Therefore, it is essential to investigate subimmunophenotypes in prostate cancer to guide potential immunotherapeutic strategies for these patients.

In the current study, we employed the non‐negative matrix factorization (NMF) algorithm to discover molecular patterns that are tightly linked to the immune infiltration of prostate cancer. Based on these patterns, three immunophenotypes were established using bulk tumor gene expression profiles from public cohorts and a real‐world AHMU‐PC cohort. Our results suggest that the immune response drives outcomes in prostate cancer, and also guiding the development of immunotherapy strategies for prostate cancer patients.

## 2 Materials and methods

### 2.1 Patient information

**Fig. 1:**
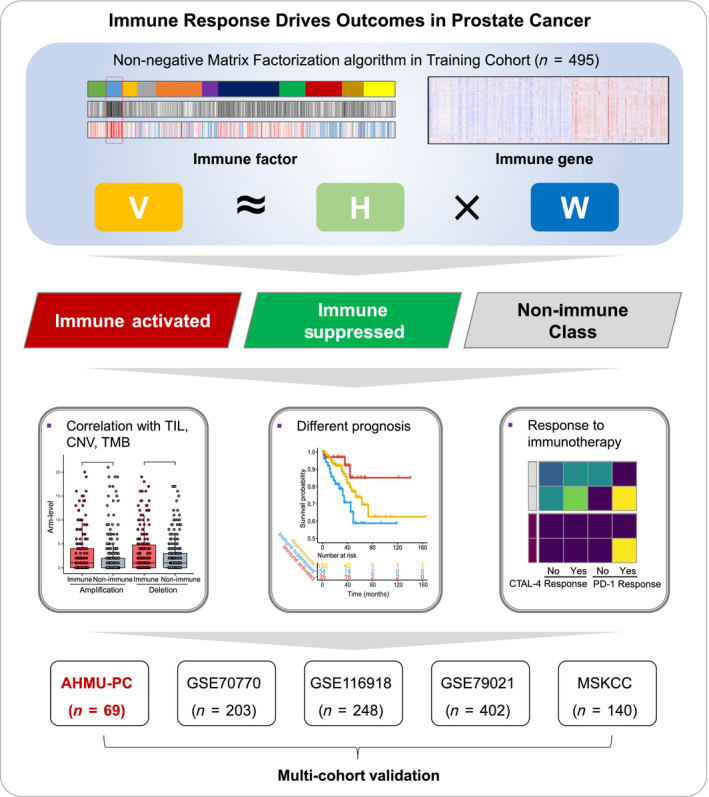
Flow chart of the current study. A total of 1,557 prostate cancer patients were analyzed, and the immunophenotypes were established based on 495 patients from the TCGA‐PRAD cohort and validated in the GSE70770, GSE116918, GSE79021, MSKCC, and AHMU‐PC cohorts. TCGA‐PRAD, The Cancer Genome Atlas‐prostate adenocarcinoma; MSKCC, Memorial Sloan‐Kettering Cancer Center.

A total of 1557 prostate cancer patients were enrolled in the current study with available gene expression profiles, clinicopathological features, and recurrence‐free survival records. The procedure of this study is depicted in Fig. 1. The Cancer Genome Atlas‐prostate adenocarcinoma (TCGA‐PRAD) cohort, which includes 495 patients, was set as the training cohort, while another four public cohorts, the Memorial Sloan‐Kettering Cancer Center (MSKCC), GSE70770, GSE116918, and GSE79021 cohorts, were set as the validation cohorts, including a total of 993 prostate cancer patients. Detailed information for all the enrolled cohorts is listed in Table 1.

**Table 1:** Summary of the clinicopathological parameters of four independent prostate cancer datasets

Parameters	TCGA‐PRAD^c^ (*n* = 495)	MSKCC^d^ (*n* = 140)	GSE70770^e^ (*n* = 203)	GSE116918^f^ (*n* = 248)	GSE79021^g^ (*n* = 402)	AHMU‐PC (*n* = 69)
Experiment type	RNA‐seq	Microarray	Microarray	Microarray	Microarray	Illumina NovaSeq
Age						
≤60	222	–	–	35	–	10
>60	273	–	–	213	–	59
Pathology T Stage^a^						
≤T2	187	86	82	127	‐	55
>T2	301	54	119	96	‐	14
Gleason score^b^						
≤7	291	117	177	141	–	37
>7	204	21	24	107	–	29
Recurrence event						
No	425	104	139	192	–	38
Yes	70	36	64	56	–	31

^a^ Seven samples lack of T stage data in TCGA database, two samples lack in GSE70770, 25 samples lack in GSE116918.

^b^ Two samples lack of Gleason score data in MSKCC, two samples in GSE70770, three samples in AHMU‐PC.

^c^ https://gdc.xenahubs.net/download/TCGA‐PRAD.htseq_fpkm.tsv.gz;

^d^ http://cbio.mskcc.org/cancergenomics/prostate/data;

^e^ https://www.ncbi.nlm.nih.gov/geo/query/acc.cgi?acc=GSE70770;

^f^ https://www.ncbi.nlm.nih.gov/geo/query/acc.cgi?acc=GSE116918;

^g^ https://www.ncbi.nlm.nih.gov/geo/query/acc.cgi?acc=GSE79021.

### 2.2 Real‐world clinical samples collection and sequencing

Moreover, we also collected formalin‐fixed, paraffin‐embedded (FFPE) samples from 69 patients with available recurrence‐free survival records from the Department of Urology, First Affiliated Hospital of Anhui Medical University (AHMU‐PC cohort). Before the FFPE sample collection, a central review of pathology was performed by an experienced pathologist. Clinicopathological characteristics were obtained from electronic records. Patients were regularly followed up by telephone, mail, or in the clinic, the endpoint of the primary outcome is the biochemical recurrence, which was defined with the presence of the PSA level greater than 0.2 ng·mL^−1^ measured 6–13 weeks after RP, followed by a confirmatory test showing a persistent PSA greater than 0.2 ng·mL^−1^ [12]. All the study designs and test procedures were performed in accordance with the Helsinki Declaration II. Ethical approval for the AHMU‐PC cohort was obtained from the Ethics Committee of the First Affiliated Hospital of Anhui Medical University (PJ2019‐09‐11). The detailed features of AHMU‐PC cohort are described in Table 1. The extraction of total RNA from FFPE samples was referring to the manufacturer’s instructions provide by RNeasy FFPE Kit (Qiagen, Germany). The quality of RNA was determined by a Nanodrop (OD260/280, Thermo Fisher) and further analyzed by Agilent 2100 bioanalyzer (Agilent). The gene expression profiles were determined by whole transcriptome sequencing based on the Illumina NovaSeq platform with a paired‐end 150‐bp sequencing strategy.

### 2.3 Bioinformatic analyses

In the TCGA‐PRAD training cohort, tumor, stromal, and immune cell transcriptome profiling data were virtually microdissected employing the unsupervised NMF method as previously described [13] through the GenePattern module ‘NMF’ [14]. The NMF algorithm, which is suitable for decomposing biological data, can factorize the gene expression matrix V (*n* genes × *m* samples) into two matrixes: a gene factor matrix* W* of (*n* genes × *k *factors) and a sample factor matrix *H* of (*m* samples × *k* factors) [15] (Fig. 1). To select immune‐related NMF factor, we employed single‐sample gene set enrichment analysis (ssGSEA, GenePattern module ‘ssGSEA’) to generate the immune score as described previously [16]. Then, the immune and nonimmune subtypes were dichotomized by the GenePattern module ‘NMFConsensus’ using the gene expression of the top 150 exemplar genes of the immune‐related NMF factor. The immune class was further divided into immune‐suppressed and immune‐activated subtypes by the nearest template prediction (NTP, GenePattern module ‘NTP’) via the activated stroma signature [17]. Manually curated gene signatures representing various immune cell types or host antitumor immunity were used to further characterize the immune classes based on the immunosuppressive or activated microenvironment via ssGSEA (Table S1). Copy number alterations (CNAs) and tumor‐infiltrating lymphocytes (TILs) were compared between different immune classes. The tumor‐infiltrating lymphocytes (TIL) abundance estimated by H&E‐stained whole‐slide images of TCGA samples was obtained from a previous study [18]. Copy number alterations (CNA) data were generated by GISTIC2.0 from GDAC Firehose (https://gdac.broadinstitute.org). We compared the differences in amplification or deletion events of both focal and arm level between immune and nonimmune classes. The neoantigen number was accessed from a previous study by Rooney *et al*. [19]. The mutation data were retrieved from TCGA (https://tcga‐data.nci.nih.gov); we calculated the number of nonsynonymous mutations per million bases to evaluate the tumor mutation burden (TMB). What’s more, we used the MutSigCV_v1.41 [20] (www.broadinstitute.org) to infer significant cancer mutated genes (*q* < 0.05) across the entire TCGA cohort with default parameters. Significantly differential mutations among the current three subtypes were further identified by the independent test with *P* < 0.05. The mutation landscape Oncoprint was drawn by R package ‘ComplexHeatmap’ [21]. To validate the immunophenotypes obtained from the training cohort, the 150 differentially expressed genes (DEGs) among the immune and nonimmune classes were used to dichotomize the subgroups into external validation cohorts with the GenePattern module ‘NMFConsensus’ and then into immune‐activated and suppressed subgroups according to the activated stromal signature. Melanoma cohort that received anti‐CTLA‐4 or anti‐PD‐1 therapy was also concerned for the immunotherapy response prediction [22]. Subclass mapping analysis (GenePattern module ‘SubMap’) was applied to detect the similarity of gene expression profile between our prostate cancer immune classifier and responders of anti‐CTLA‐4 or anti‐PD‐1 in the melanoma cohort.

### 2.4 Immunohistochemistry (IHC) staining for CD163 and α‐SMA

IHC staining was used to validate the immunophenotypes in the AHMU‐PC cohort. CD163 (Anti‐CD163 antibody: Cat. ab182422, Abcam Inc., Cambridge, MA, USA) was chosen as the cell marker for macrophages, while α‐SMA (anti‐α‐SMA antibody: Cat. Ab7817, Abcam Inc., Cambridge, MA, USA) was employed to reflect stromal activation and distinguish the immune‐activated and immune‐suppressed subtypes. The detailed steps of the IHC procedure have been previously reported [23, 24]. α‐SMA is universally expressed in stromal cells, and we used the positively stained region score (0, negative; 1, 1%–10; 2, 11–50%; 3, 51–80%; and 4, >80% positive area) multiplied by the immunostaining intensity score (0, no staining; 1, weak; 2, mild; and 3, strong intensity) to semiquantify the results. For the CD163 staining, we directly used ImageJ software (NIH, Bethesda, USA) to count positively stained cells [25].

### 2.5 Statistical analysis

Comparisons of continuous data (TIL abundance, CNV, TMB, neoantigens, and signature score) between two immune molecular subtypes were performed by t‐test and Wilcoxon rank‐sum test for normal and non‐normal distribution data, respectively. Kaplan–Meier plots and log‐rank tests were employed to perform survival analysis among three immunophenotypes for recurrence‐free survival. Correlations between immune molecular classification and proposed molecular subtypes were analyzed by the chi‐square test. A two‐sided *P*‐value < 0.05 was considered statistically significant. All analyses were performed by genepattern [14] and r version 4.0.2 (http://www.r‐project.org).

## 3 Results

### 3.1 Discovering immune‐related factor and identifying the immune subclasses of prostate cancer

**Fig. 2:**
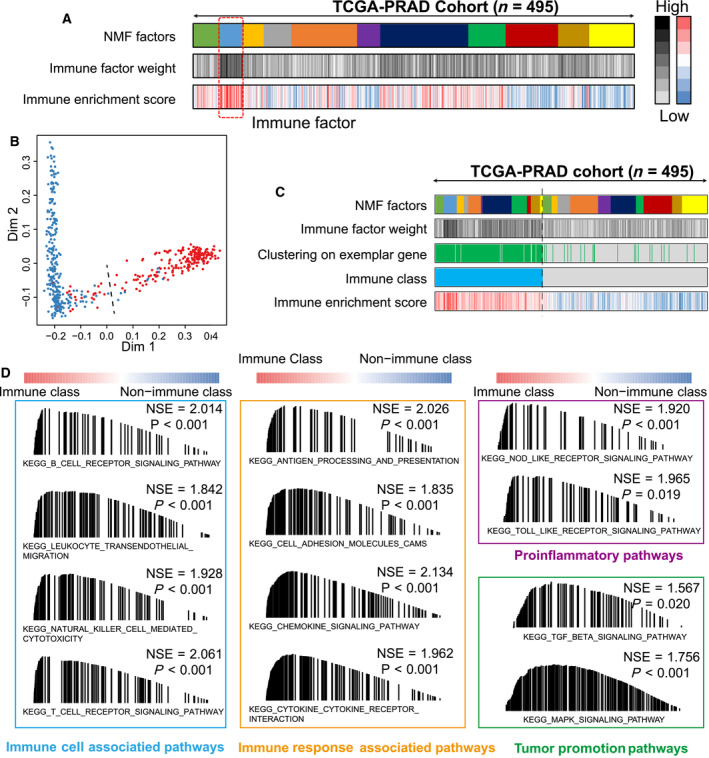
Identification of the immune‐related clustering factor by non‐negative matrix factorization (NMF) analysis. (A) 11 clustering factors obtained from NMF analysis, with the second factor enriched the most patients with high immune enrichment scores. (B) The immune and nonimmune classes were adjusted by the multidimensional scaling (MDS) random forest analysis, via the expression matrix of the top 150 exemplar genes. (C) Heatmap showing the distribution of patients in different NMF factors, immune factor weight, exemplar genes‐based clustering, immune enrichment score, and final immune classes. (D) Gene Set Enrichment Analysis (GSEA) results showing the activated signaling pathways in the immune class.

A total of 1,557 prostate cancer patients were enrolled in the current study (Table 1) with available gene expression profiles, clinicopathological features, and recurrence‐free survival records. The procedure of this study is depicted in Fig. 1. The NMF algorithm was first employed to conduct a virtual microdissection of the gene expression profiles of 495 prostate cancer patients derived from the training TCGA‐PRAD cohort. The second factor of the eleven expression patterns (NMF clusters) was of immunologic relevance and had a relatively higher immune enrichment score than the others (Fig. 2A); therefore, we termed this NMF factor the ‘immune factor’. We chose the top 150 weighted genes as exemplar genes representing the second immune factor (Table S2). We performed Gene Ontology enrichment analysis and found that the 150 exemplar genes were most enriched in T‐cell activation, leukocyte migration, and lymphocyte differentiation pathways (Figure S1); further, the top five exemplar genes showed positive relationships with B cells, CD8 + T cells, CD4 + T cells, macrophages, neutrophils, and dendritic cells (all *P* < 0.05, Figure S2).

**Fig. 3:**
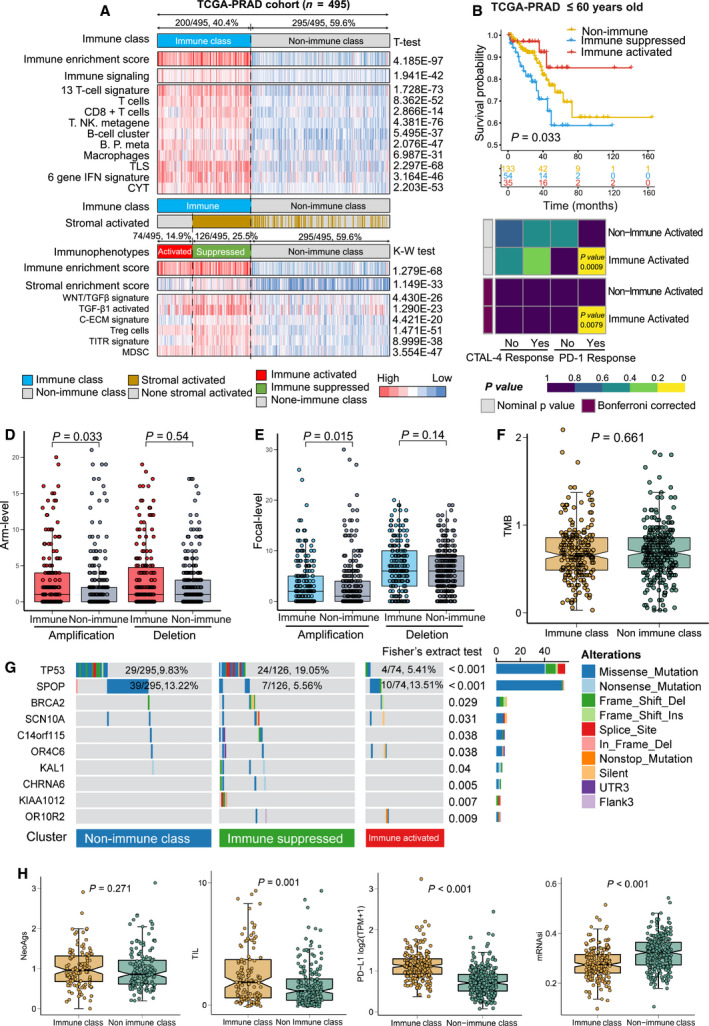
Identification of the immunophenotypes among the TCGA‐PRAD cohort, and comparing their differences at tumor‐infiltrating lymphocytes, copy number alterations, gene mutations, neoantigens, tumor stemness, and PD‐L1 expression levels. (A) Consensus‐clustered heatmap by the exemplar genes of NMF selected immune factor and refined by multidimensional scaling random forest to define the immune class (200/495, 40.4%, sky‐blue bar); nearest template prediction (NTP) using a signature capturing activated stroma identified immune‐suppressed (126/495, 25.5%; light‐green bar) and immune‐activated (74/495, 14.9%; red bar) classes; in the heat map, high and low single‐sample gene set enrichment scores are represented in red and blue, respectively. Positive prediction of activated stroma signature as per NTP is indicated in brown and its absence is in gray; (B) different recurrence‐free survival in three immunophenotypes among patients less or equal to 60 years old in TCGA‐PRAD cohort; (C) subclass mapping analysis manifested that patients with immune‐activated subtype were more likely to respond to anti‐PD‐1 treatment (Bonferroni‐corrected *P*‐value = 0.0079); (D) arm‐level copy number amplification and deletion; (E) focal‐level copy number amplification and deletion; (F) tumor mutant burden difference; (G) differentially mutated genes among three immune subgroups (some patients in nonimmune class without gene mutations hided); (H) neoantigens difference; (I) tumor‐infiltrating lymphocytes difference; (J) PD‐L1 expression difference; (K) tumor stemness difference represented by the mRNAsi. The comparison between two groups was conducted by Student’s t‐test. TCGA‐PRAD, The Cancer Genome Atlas‐prostate adenocarcinoma; CYT, cytolytic activity score; TITR, tumor‐infiltrating Tregs; MDSC, myeloid‐derived suppressor cell; TLS, tertiary lymphoid structure; C‐ECM, cancer‐associated extracellular matrix. t‐Test, Student’s t‐test, K‐W test, Kruskal–Wallis test.

Consensus clustering based on the 150 exemplar genes was performed on all 495 patients, and a multidimensional scaling (MDS) random forest was employed to further divide patients into immune and nonimmune subclasses (Fig. 2B,C). We compared the differences in the activated signaling pathways between the immune and nonimmune classes by GSEA and found that immune cell‐associated pathways, immune response pathways, proinflammatory pathways, and tumor promotion pathways were significantly activated in the immune class [all of which had false discovery rate (FDR) <0.05; Fig. 2D]. Moreover, patients belonging to the immune class showed significantly higher enrichment scores for immune signals than those in the nonimmune class, including T cell‐, B cell‐, NK cell‐, and macrophage‐associated signatures, as well as tertiary lymphoid structure (TLS), cytolytic activity score (CYT), and IFN signatures (all *P* < 0.05, Fig. 3A upper panel). Taken together, the results shown in Fig. 2 and the upper panel of Fig. 3A indicate that the identified immune‐related factors and exemplar genes are able to define the immune subclasses in prostate cancer.

### 3.2 Two distinct immunophenotypes highlighted by different microenvironmental conditions

Several studies have revealed the heterogeneity of the immune microenvironment in tumors. The different infiltration statuses of Treg cells and myeloid‐derived suppressor cells are correlated with divergent responses to anti‐PD‐1 immunotherapy; these tumors are defined as immune ‘hot’ or ‘cold’ groups, respectively [26]. Therefore, we sought to explore the subimmunophenotypes of the immune class.

According to previous reports, the activated stromal response is negatively associated with immune activation, and we found that 63.0% (126/200) of patients in the immune class were characterized by high stromal enrichment scores (Fig. 3A, lower panel). TGF‐β is regarded as the central mediator of immune suppression in the immune microenvironment [27], and the high levels of extracellular matrix cytokines (C‐ECM) induced by activated cancer‐associated fibroblasts are able to recruit immune suppressive cells [28]. In line with our expectations, we found that the signatures of WNT/TGF‐β, TGF‐β1, and C‐ECM were more highly enriched in the stromal‐activated subgroup (termed the immune‐suppressed subtype) than in the nonimmune class (all *P* < 0.05, Fig. 3A, lower panel), and the remaining 37.0% of patients (74/200) belonged to the immune‐activated subtype. Furthermore, we observed increased expression of IL‐11, TGFB1, and TGFB2 in the immune‐suppressed subtype compared with the immune‐activated subtype (all *P* < 0.05, Fig. S3), a result consistent with a previous publication [29]. A recent study suggests that PAK4 is enriched in nonresponding tumor biopsies [30], and we also observed that PAK4 was significantly more highly expressed in the immune‐suppressed subtype than in the immune‐activated subtype (*P* = 0.037, Fig. S3). In addition, we found that the tumor‐infiltrating Treg (TITR) signature (*P* < 0.01) and Treg cell signature (*P* = 0.017) were mostly enriched in the immune‐suppressed subtype (Fig. 3A, lower panel, Table 2), while the Th17 cell infiltration signature was significantly enriched in the immune‐activated subtype (*P* = 0.034, Fig. S3). Taken together, the results shown in Fig. 3A and Fig. S3 define two distinct immunophenotypes, the immune‐suppressed and immune‐activated subclasses, based on tumor microenvironmental activities.

**Table 2:** The proportion of three immune subtypes in six enrolled cohorts. MSKCC, Memorial Sloan‐Kettering Cancer Center; n, number; TCGA‐PRAD, The Cancer Genome Atlas‐Prostate Adenocarcinoma

Cohort	TCGA‐PRAD	GSE70770	GSE116918	MSKCC	GSE79021	AHMU‐PC
Number, n	495	203	248	140	402	69
Immune activated, n (%)	74 (14.9%)	42 (20.7%)	33 (13.3%)	34 (24.3%)	87 (21.6%)	14 (20.3%)
Immune suppressed, n (%)	126 (25.5%)	56 (27.6%)	75 (30.2%)	33 (23.6%)	75 (18.7%)	19 (27.5%)
Nonimmune class, n (%)	295 (59.6%)	105 (51.7%)	140 (54.5%)	73 (52.1%)	240 (59.7%)	36 (52.2%)

### 3.3 Immune activation is linked to favorable recurrence‐free survival and anti‐PD‐1 immunotherapy

The clinicopathological features are important evaluation criteria to demonstrate the malignant degree of prostate cancer. Here, we explored the distributions of the three immunophenotypes according to their different clinicopathological features. Most patients in the immune‐suppressed class were in the advanced stage compared to the other two subclasses in the TCGA‐PRAD cohort [Gleason score > 7 (48/126, 61.9%, *P* < 0.001), T stage > T2 (99/125, 79.2%, *P* < 0.001)] (Fig. S4). The different recurrence‐free survival outcomes according to the immune molecular subgroups were also assessed. In the TCGA‐PRAD cohort, among patients younger than 60 years old, we observed that the immune‐activated subtype showed favorable recurrence‐free survival, while the immune‐suppressed subtype showed poor recurrence‐free survival, and the nonimmune class showed a moderate recurrence outcome (*P* = 0.033, Fig. S3B). We tested the potential capacity of the immune molecular classification system to select candidate patients to receive anti‐PD‐1/PD‐L1 immunotherapy. SubMap analysis indicated that patients in the immune‐activated subtype shared a similar gene expression profile to melanoma patients who were responsive to anti‐PD‐1 immunotherapy (Bonferroni‐corrected *P* = 0.0079, Fig. 3C). In summary, according to the results shown in Fig. 3B,C and Fig S4, patients in the immune‐activated class showed the best recurrence‐free survival outcomes and might benefit more from anti‐PD‐1/PD‐L1 immunotherapy than other patients.

### 3.4 ***Correlations between immune class and copy number alterations, tumor*** *‐* ***infiltrating lymphocyte enrichment, and reduced cancer stemness***

Somatic mutations in tumor cells are a double‐edged sword in malignant tumors; these mutations can promote tumorigenesis or they can be recognized by the immune system and lead to forcefully acquired immunity. The immunogenicity of the antitumor immune response is based on the non‐self‐antigen, which is generated by somatic mutations [31]. Neoantigens can be captured by antigen‐presenting cells and then induce the activation of neoantigen‐specific T cells; subsequently, tumor cells are killed by tumor‐infiltrating lymphocytes (TILs) through recognition of the neoantigen [32].

In the TCGA cohort, the immune class showed a high burden of amplification at both the arm and focal levels (*P*_Arm‐Amp_ = 0.033, *P*_Focal‐Amp_ = 0.015) instead of deletion (*P*_Arm‐del_ = 0.54, *P*_Focal‐del_ = 0.14) (Fig. 3D,E). Furthermore, we found that copy number alterations (CNAs) of several immune checkpoints, *PD‐1*, *PD‐L1*, *LGALS9*, and *CD48*, were positively associated with the infiltration of immunocytes (Fig. S5). Regarding TMB and neoantigens, no differences were observed between the immune and nonimmune classes (*P*_TMB_ = 0.661, Fig. 3F, *P*_NeoAgs_ = 0.271, Fig. 3H).

Notably, we revealed a different mutation landscape among the three immunophenotypes based on MutSigCV algorithm analysis (Fig. 3G, Table S3). Specifically, the mutation frequency of TP53 in the immune class were higher than that in the nonimmune class (14.00% vs. 9.83%), particularly in the immune‐suppressed subtype (19.05%, Fisher’s extract test, *P* < 0.001). Regarding SPOP, fewer mutations were observed in the suppressed subtype than in both the immune‐activated subtype and the nonimmune class (5.56% vs. 13.51% and 13.22%, *P* < 0.001). In addition, we identified several immune class‐specific mutated genes, including *BRCA2, SCN10A, C14orf115, OR4C6, KAL1, CHRNA6, KIAA1012, and OR10R2* (all *P* < 0.05). Some of these genes have already been used in clinical tests. The gene mutation signatures of each immunophenotype are shown in Fig. S6.

Regarding TILs, we found a significantly higher density of TILs in the immune class than in the nonimmune class (*P* = 0.001, Fig. 3I). The expression of PD‐L1 was also increased, along with a greater infiltration of CD8 + T cells, in the immune class than in the nonimmune class (*P* < 0.001, Fig. 3J), consistent with a previous study [33]. Moreover, Miranda *et al*. [34] reported a negative association between stemness and the immune response and revealed that it is not readily attributable to a low neoantigen load. Here, we revealed reduced stemness, represented by mRNAsi [35], in the immune class compared to the nonimmune class (*P* < 0.001, Fig. 3K). Taken together, the results shown in Fig. 3, Figs S5 and S6, and Table S3 reveal that the immune class is correlated with significantly higher CNAs and higher TIL enrichment but not with TMB and neoantigens.

### 3.5 Reappearance of the three immunophenotypes in a real‐world AHMU‐PC cohort

To confirm the accuracy of the NMF algorithm and activated stromal signature‐based immunophenotypes, a total of 150 upregulated genes were identified between the immune and nonimmune classes (Table S4) as the immune classifier to distinguish these classes. The top five DEGs showed positive relationships with B cells, CD8 + T cells, CD4 + T cells, macrophages, neutrophils, and dendritic cells (all *P* < 0.05, Fig. S7), which indicated that these genes could reflect immunocyte infiltration effectively.

**Fig. 4:**
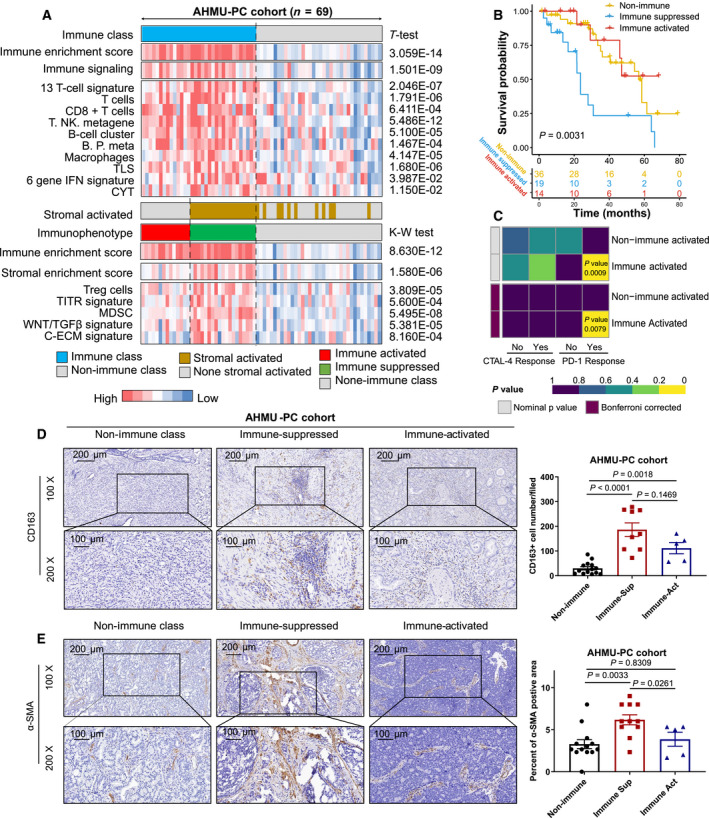
Successful validation of the immunophenotypes in the AHMU‐PC cohort. (A) Heatmap showing the different enrichment of characteristic signatures among immune‐activated, immune‐suppressed, and nonimmune groups; (B) Kaplan–Meier plot showing the recurrence‐free survival outcome in three immunophenotypes; (C) subclass mapping analysis manifested that patients with immune‐activated subtype were more likely to respond to anti‐PD‐1/PD‐L1 treatment (Bonferroni‐corrected *P*‐value = 0.0399); immunohistochemistry staining and quantification of CD163 (D) and α‐SMA (E) in prostate cancer patients with different immune status (nonimmune, immune‐suppressed, and immune‐activated classes) from AHMU‐PC cohort, Scale bar, 200 μm, 100 μm. *t*‐Test, Student’s *t*‐test, K‐W test, Kruskal–Wallis test.

In the AHMU‐PC cohort, we retrospectively collected FFPE tissue of 69 patients with available clinicopathological features and performed long‐term follow‐up and RNA sequencing to obtain the gene expression profile (Table S5). With the help of the NMF consensus pattern, 47.8% (33/69) of patients had a high immune enrichment score and were assigned to the immune class, while the other 52.2% of patients belonged to the nonimmune class. Furthermore, the immune class was subsequently classified into immune‐activated (14/69, 20.3%) and suppressed (19/69, 27.5%) subtypes (Table 2). Similar to the results obtained above, patients in the immune class showed higher enrichment scores for the T cell, B cell, macrophage, TLS, CYT, and IFN signatures (all *P* < 0.05) than those in the nonimmune class. The immune‐suppressed subtype displayed high scores for the stromal enrichment score, TITR, MDSC, and C‐ECM signatures (all *P* < 0.05, Fig. 4A). We also employed Kaplan‐Meier analysis to determine the recurrence‐free survival differences among the three immunophenotypes. Consistently, the immune‐suppressed subtype showed a worse recurrence‐free survival outcome than the immune‐activated and nonimmune subgroups (*P* = 0.0083, Fig. 4B). Furthermore, patients in the immune‐activated subtype were mostly enriched at the early pathological stage, as assessed by the Gleason score (92.3% vs. 44.4%, 48.57%, Kruskal–Wallis test, *P* = 0.0127) and pathology T stage (92.9% vs. 65.0%, 75.0%, Kruskal–Wallis test, *P* = 0.368, Fig. S8). Patients in the immune‐activated subgroup of the AHMU‐PC cohort seemed to benefit more from anti‐PD‐1/PD‐L1 immunotherapy than patients in the non‐immune‐activated class (Bonferroni‐corrected *P* = 0.0399, Fig. 4C).

To confirm the accuracy of the classification system, we employed immunohistochemistry (IHC) staining for samples from the AHMU‐PC cohort (tissue sections were obtained from the 69 patients mentioned above). CD163 is a marker of macrophages that was used to distinguish the immune and nonimmune classes in this study, while the stromal marker α‐SMA was used to discriminate the immune‐activated and immune‐suppressed subgroups. We obtained IHC results consistent with the results derived from the NMF‐based immune molecular classifier. We observed increased CD163 + cells in both the immune‐suppressed (*P* < 0.0001) and immune‐activated (*P* = 0.0018) subtypes compared to the nonimmune class (Fig. 4D). Regarding the IHC staining of α‐SMA, we observed a higher H‐score in the immune‐suppressed subtype than in both the immune‐activated subtype (*P* = 0.0033) and nonimmune class (*P* = 0.0261) (Fig. 4E). Taken together, the results shown in Fig. 4 and Figs S7 and S8 validate the three immunophenotypes in the AHMU‐PC cohort and confirm the consistency of the RNA‐sequence‐based immunophenotypes and real IHC staining findings. Patients in the immune‐suppressed group showed the worst recurrence‐free survival outcomes, while patients in the immune‐activated subtype might benefit from anti‐PD‐1/PD‐L1 therapy.

### 3.6 Validation of the three immunophenotypes in external cohorts

Moreover, we recruited an additional 993 prostate cancer patients with available gene expression profiles and matched clinicopathological features (Table 1) for validation. For the results of immune signatures in these four external validation cohorts, the immune enrichment score and immune signaling signature were significantly enriched in the immune class (all *P* < 0.05), as well as the ssGSEA results of the T cell, B cell, macrophage, TLS, CYT, and IFN signatures (all *P* < 0.05). The activated and suppressed subtypes were divided by the stromal activation signature generated from the nearest template prediction (NTP) method, and the immune‐suppressed subtype showed a higher SES than the immune‐activated subtype in these three external cohorts (all *P* < 0.05). Furthermore, higher enrichments of the Treg cell, TITR, MDSC, WNT/TGFβ, and C‐ECM signatures were identified in the immune‐suppressed subtype than in the immune‐activated subtype in these three external cohorts (all *P* < 0.05).

**Fig. 5:**
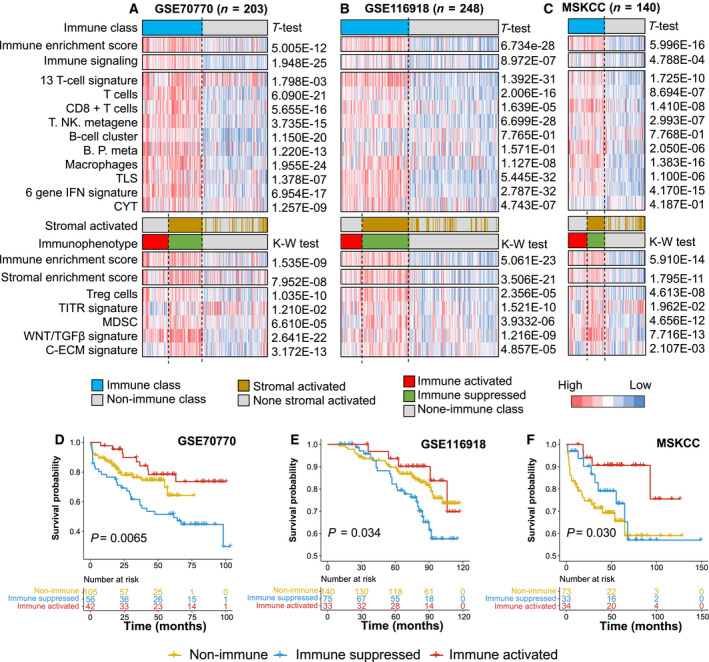
Immunophenotypes associated with the different recurrence‐free survival outcomes of prostate cancer patients. (A–C) Consensus‐clustered heatmap by the exemplar genes of NMF selected immune factor and refined by multidimensional scaling random forest to define the immune class; nearest template prediction (NTP) using a signature capturing activated stroma identified two distinct immune response subtypes: immune‐suppressed and immune‐activated classes; in the heat map, high and low single‐sample gene set enrichment scores are represented in red and blue, respectively. Positive prediction of activated stroma signature as per NTP is indicated in brown and its absence is in gray; (D) different recurrence‐free survival of three immunophenotypes in GSE70770 cohort; (E) different recurrence‐free survival of three immunophenotypes in GSE116918 cohort; (F) different recurrence‐free survival of three immunophenotypes in MSKCC cohort. MSKCC, Memorial Sloan‐Kettering Cancer Center. t‐Test, Student t‐test, K‐W test, Kruskal–Wallis test.

In the GSE70770 cohort, 51.7% (105/203) of patients were classified as the nonimmune subtype, 42 patients (20.7%) were classified as the immune‐activated subtype, and 56 patients (27.6%) were classified as the immune‐suppressed subtype (Fig. 5A, Table 2). Of the 248 patients from the GSE116918 cohort, 54.5% (140/248) belonged to the nonimmune subtype, 75 patients (30.2%) belonged to the immune‐suppressed subtype, and the other 33 (13.3%) belonged to the immune‐activated subtype (Fig. 5B, Table 2). In the MSKCC cohort, 73 patients (52.1%) were assigned to the nonimmune subtype, while 34 (24.3%) and 33 (23.6%) were assigned to the activated and suppressed subtypes, respectively (Fig. 5C, Table 2). Another 402 patients extracted from the GSE79021 cohort also displayed similar results: 21.6% (87/402) of patients belonged to the immune‐activated subtype, 18.7% (75/402) belonged to the immune‐suppressed subtype, and the remaining 240 belonged to a nonimmune class (Fig. S9, Table S2).

Notably, consistent with the results obtained above, in these three external validation cohorts, patients belonging to the immune‐activated subtype showed the best recurrence‐free survival, while patients belonging to the nonimmune subtype showed the worst recurrence‐free survival, and the immune‐suppressed group showed an in‐between outcome (Fig. 5D–F). Taken together, the results shown in Fig. 5 and Fig. S9 prove the stability and significance of this newly established immune molecular‐based classification system in prostate cancer.

### 3.7 Comparison between the newly defined immunophenotypes and previously established molecular features

We also sought to integrate the immunophenotypes with previously established immune molecular features. Thorsson *et al*. [36] generated a six‐subtype immune molecular feature, including wound healing, IFN‐γ‐dominant, inflammatory, lymphocyte depleted, immunologically quiet, and TGF‐β‐dominant features. We revealed that most prostate cancer patients belonged to the inflammatory group, and the immune‐activated subtype ranked as the highest proportion of the inflammatory group (46/50, 92.0%), followed by the immune‐suppressed subtype (85/114, 74.6%) and nonimmune class (176/241, 73.0%) (*P* < 0.001, Fig. S10). Zhao *et al*. [37] defined the molecular subtypes of pan‐cancer using the PAM50 classifier, which was approved by the FDA for clinical prognostic evaluation of breast cancer [38]. We classified the 495 patients in the TCGA‐PRAD cohort into luminal A, luminal B, and basal‐like subgroups. We revealed that the immune‐activated subtype contained more luminal A‐like patients, while the immune‐suppressed subtype contained more luminal B‐like patients (*P* < 0.001, Fig. S10). Tamborero *et al*. [39] provided a comprehensive landscape of the immune characteristics of solid tumors. We compared the patient distributions of our study and Tamborero *et al*.’s study and revealed that the immune subtypes identified in our study were similar to theirs. Patients in the immune‐activated subtype belonged to the higher cytotoxic cell groups (Groups 3–6), while patients in the nonimmune class was consistent with the lower cytotoxic cell infiltrated groups (Group 1 and Group 2) (Fig. S11). Taken together, these results establish a novel immune feature‐based classification system that is able to predict recurrence‐free survival of prostate cancer patients, and the patients in the immune‐activated subgroup seem to be more responsive to anti‐PD‐1/PD‐L1 immunotherapy than other patients.

## 4 Discussion

CRPC patients face more severe symptoms and complications than other early‐stage patients, including a reduced survival time, more bone metastasis‐induced bone pain, greater spinal cord compression, more extensive ureteric obstruction, and more renal failure [40]. Several therapeutic agents have been approved for the treatment of CRPC, including immune‐associated sipuleucel‐T; yet, the potential treatment of anti‐PD‐1/PD‐L1 is still under clinical evaluation [41]. Immune checkpoints promote or inhibit factors in the TME, and several immune checkpoints can help tumors escape recognition and attack the host immune system [42, 43]. Anti‐PD‐1/PD‐L1 therapy has been utilized for several malignant tumors but can only offer benefits to some patients. In the IMvigor210 trial, only 27.4% (68/248) of bladder cancer patients benefited from treatment with atezolizumab (a PD‐L1 inhibitor). For gastric tumors, only 11.6% of enrolled patients responded to pembrolizumab monotherapy in the KEYNOTE‐059 trial, and the objective response rate of nivolumab in the ATT RAC TION‐2 trial was only 11.2% [44, 45]. Therefore, it is essential to comprehensively describe the prostate cancer immune microenvironment, which will help to identify suitable patients to undergo immunotherapy.

The NMF approach is a virtual separation approach that has been applied successfully in several fields, including image and pattern recognition, signal processing, and text mining [46], and has obtained novel insights into cancer type discovery based on gene expression profiles by identifying exemplar genes [17]. In the current study, we proposed a robust immunogenomic classification system for prostate cancer based on the NMF algorithm. The immune exemplar genes and stromal activation signature enabled patient stratification into three immunophenotypes: immune‐activated, immune‐suppressed, and nonimmune classes. A similar method to reveal the immunophenotypes was applied and validated in hepatocellular carcinoma, gastric cancer, and head and neck squamous cell carcinoma [47, 48, 49]. Initially, we observed the landscape of immune class distributions in prostate cancer derived from the TCGA‐PRAD cohort. Of the 495 patients, 40.4% belonged to the immune class; patients in this class exhibited greater enrichment of immunocytes, cytolytic activity, and IFN signaling than patients in the nonimmune class, and these signatures were also similar to the signatures observed in patients responding to immunotherapy [19, 50]. Subsequently, we dissected the immune class into immune‐activated and immune‐suppressed subtypes based on the stromal‐activated signature. Overall, 14.9% of patients belonged to the immune‐activated subtype and had lower enrichment of the stromal enrichment score, WNT/TGF‐β, C‐ECM, and TITR signatures, while the remaining 25.5% were immune suppressed. Similar immunophenotypes were also validated in four external cohorts. The immune‐activated subtype comprised 20.7% of the GSE70770 cohort, 13.3% of the GSE116918 cohort, 24.3% of the MSKCC cohort, 21.6% of the GSE79021 cohort, and 20.3% of the AHMU‐PC cohort. These results indicate that only approximately 13.3–24.3% of overall patients could benefit from immunotherapy.

The PAM50 classifier was first used to subclassify breast cancer into four subtypes: luminal A, luminal B, HER2‐enriched, and basal‐like. The FDA approved the application of the PAM50 classifier in the clinical prognostic evaluation of breast cancer patients in 2013 [38]. PAM50 subtypes also display different prognostic outcomes and responses to clinical therapy among bladder cancer patients [51]. As a supplement to Zhao *et al*.’s [37] work on the PAM50 classifier and its application to prostate cancer patients, we classified the 495 patients in the TCGA‐PRAD training cohort into luminal A, luminal B, and basal‐like subgroups. After comparing the distributions of the three immunophenotypes and PAM50 subtypes, we revealed that the immune‐activated class contained more luminal A‐like patients, while the immune‐suppressed subtype contained more luminal B‐like patients. These results are consistent with Zhu *et al*.’s work [52], demonstrating that luminal A patients showed higher expression of immune checkpoint genes (PD‐L1 and CTLA‐4) and chemokine genes (CXCL9 and CXCL10). Recently, Thorsson *et al*. [36] also generated the pan‐cancer atlas of TCGA, which identified six pan‐cancer immune cells. Of the prostate cancer patients evaluated in this study, most belonged to the inflammatory subtype, and the distributions were similar between the immune and nonimmune classes. Interestingly, after dividing the immune class into activated and suppressed subtypes, the inflammatory subtype accounted for the majority of immune‐activated patients (92.0%). The inflammatory subtype is characterized by elevated Th17 cells [36], and we also revealed increased infiltration of Th17 cells in the immune‐activated subtype. Derhovanessian *et al*. [53] reported that Th17 cells were higher in patients who were responsive to immunotherapy than in nonresponders and were negatively correlated with tumor stage.

The CNAs were decreased compared with the nonimmune class at both the arm and focal levels, as reported in an immunophenotype study in gastric cancer and head and neck carcinoma [47, 48]. However, we observed a different phenomenon in which the arm level of CNA in the immune‐activated subtype was increased, which might be linked to the elevated infiltration of immunocytes, the increased release of cytokines, and the CNA of immune checkpoint genes in prostate cancer [54]. No differences in terms of TMB and neoantigens were found between the immune and nonimmune classes in our study. Although the somatic mutation frequencies of prostate cancer are dramatically lower than those in melanoma [55], Subudhi *et al*. [56] reported that some metastatic castration‐resistant prostate cancer patients who received ipilimumab treatment can still benefit from immunotherapy, with a median number of nonsynonymous somatic mutations of 76.

We presented the gene somatic mutation landscape in the three immunophenotypes. Mutations in TP53 were mostly observed in the nonimmune class, and the proportion was only 5.4% in the immune‐activated subtype. Jiang *et al*. [57] demonstrated that the TP53 mutation results in depressed immune activity in gastric cancer, and less active immune pathways and cell types were observed in TP53‐mutated gastric cancer patients. Carlisle *et al*. [58] also reported that the TP53 mutation was correlated with the poor efficacy of immunotherapy after adjusting for PD‐L1 expression in NSCLC. There were more mutations in SPOP in the immune‐activated subtype than in the immune‐suppressed subtype in our study. Zhang *et al*. [59] demonstrated that SPOP promotes ubiquitin‐mediated degradation of PD‐L1, and mutant SPOP leads to elevated PD‐L1 levels in prostate cancer patients.

The novel three defined immunophenotypes are essential for selecting suitable immunotherapies for prostate cancer patients. Patients in the immune‐activated subtype could benefit more from single ICB treatment, while immune‐suppressed patients could benefit from TGF‐β inhibitors plus ICB therapy. Likewise, the fusion protein M7824, comprising TGF‐β Trap linked to the C terminus of the human anti‐PD‐L1 heavy chain, is more suitable for immunosuppressed patients than other patients, as it decreases TGFβ‐induced signaling and promotes the activation of CD8 + T cells and NK cells [60]. For the nonimmune class, the combination of anti‐CTLA‐4 and anti‐PD‐1/PD‐L1 therapy, which attracts the infiltration of immune cells in the TME and maintains their activated status, might aid in stimulating a response in nonresponders [61].

## 5 Conclusion

We establish and validate a novel immune subtype classifier based on the expression profiles of 1,557 prostate cancer patients, including 69 real‐world PCa patients from our center. Patients in the immune‐activated subtype might benefit more from anti‐PD‐1/PD‐L1 therapy. Our findings suggest that the immune response drives outcomes in prostate cancer, which offers inspiration for the development of immunotherapy for prostate cancer patients in the future.

## Conflict of interests

The authors have declared no conflicts of interest.

## Ethics approval

The patient data in this work were acquired from publicly available datasets for which the informed consent of each patient was complete. For the AHMU‐PC cohort, the research contents and programs were reviewed and approved by the Ethics Committee of the First Affiliated Hospital of Anhui Medical University (PJ‐2019‐09‐11), and patient consent for the retrospective cohorts was waived. The study methodologies conformed to the standards set by the Declaration of Helsinki.

## Authors contributions

Jialin Meng, Meng Zhang, and Chaozhao Liang involved in conception and design. Yujie Zhou, Xiaofan Lu, Zichen Bian, and Jun Zhou performed the collection and assembly of data. Yujie Zhou, Xiaofan Lu, Yiding Chen, Li Zhang, and Zongyao Hao analyzed and interpreted the data. Jialin Meng, Meng Zhang, and Yujie Zhou wrote the manuscript. All the authors finally approved the manuscript.

## Supporting information


**Fig S1.** Pathway enrichment of the top 150 exemplar genes.
**Fig S2.** The association between the infiltration of immunocytes and the top 5 exemplar genes of immune factor.
**Fig S3.** The different expressions of stromal markers and infiltration of Th17 cells in immune activated and suppressed classes.
**Fig S4.** The distribution of Gleason score, PSA, Age and pathological T stage among three immunophenotypes in TCGA‐PRAD, MSKCC, GSE116918 and GSE70770 cohorts.
**Fig S5.** The association between copy number variation of immune checkpoints and immunocyte infiltration.
**Fig S6.** The mutational landscape showed the top mutated genes in nonimmune, immune‐activated, and immune‐suppressed subgroups in TCGA‐PRAD cohort.
**Fig S7.** The association between the infiltration of immunocytes and the top 5 differentially expressed genes among immune and nonimmune classes.
**Fig S8.** The distribution of clinicopathological features among three immunophenotypes in AHMU‐PC cohort.
**Fig S9.** Successful validation of the immunophenotypes among the GSE79021 cohort.
**Fig S10.** Association of the three immunophenotypes with the six pan‐cancer immune molecular subgroups.
**Fig S11.** Association of the three immunophenotypes with the six molecular subgroups defined by Tamborero’s study displayed by Sankey plot.

**Table S1.** Immune associated gene signatures used in this study.
**Table S2.** Top 150 weighted genes of factor 2.
**Table S3.** Differentially mutated genes based on immune molecular subtypes.
**Table S4.** Top 150 differentially expressed genes in immune class compare with non‐immune class in the TCGA training cohort.

**Table S5.** Detailed immunophenotype, immune enrichment score, stromal stromal enrichment score and clinical features of AHMU‐PC cohort.

**Appendix S1.** Materials and Methods.

## Data Availability

All data used in this work can be acquired from the GeneExpression Omnibus (GEO; https://www.ncbi.nlm.nih.gov/geo/) and the GDC portal (https://portal.gdc.cancer.gov/). Data from the AHMU‐PC cohort are available from the corresponding author through reasonable request.
