# Supplementary material for: Immune response drives outcomes in prostate cancer: implications for immunotherapy
Source: Mol Oncol. 2020 Dec 29;15(5):1358–75. doi: 10.1002/1878-0261.12887 (PMC8096785; doi:10.1002/1878-0261.12887)
Supplement: Supplementary file 1 — Fig S1. Pathway enrichment of the top 150 exemplar genes. Fig S2. The association between the infiltration of immunocytes and the top 5 exemplar genes of immune factor. Fig S3. The different expressions of stromal markers and infiltration of Th17 cells in immune activated and suppressed classes. Fig S4. The distribution of Gleason score, PSA, Age and pathological T stage among three immunophenotypes in TCGA‐PRAD, MSKCC, GSE116918 and GSE70770 cohorts. Fig S5. The association between copy number variation of immune checkpoints and immunocyte infiltration. Fig S6. The mutational landscape showed the top mutated genes in nonimmune, immune‐activated, and immune‐suppressed subgroups in TCGA‐PRAD cohort. Fig S7. The association between the infiltration of immunocytes and the top 5 differentially expressed genes among immune and nonimmune classes. Fig S8. The distribution of clinicopathological features among three immunophenotypes in AHMU‐PC cohort. Fig S9. Successful validation of the immunophenotypes among the GSE79021 cohort. Fig S10. Association of the three immunophenotypes with the six pan‐cancer immune molecular subgroups. Fig S11. Association of the three immunophenotypes with the six molecular subgroups defined by Tamborero’s study displayed by Sankey plot. [file MOL2-15-1358-s001.docx]

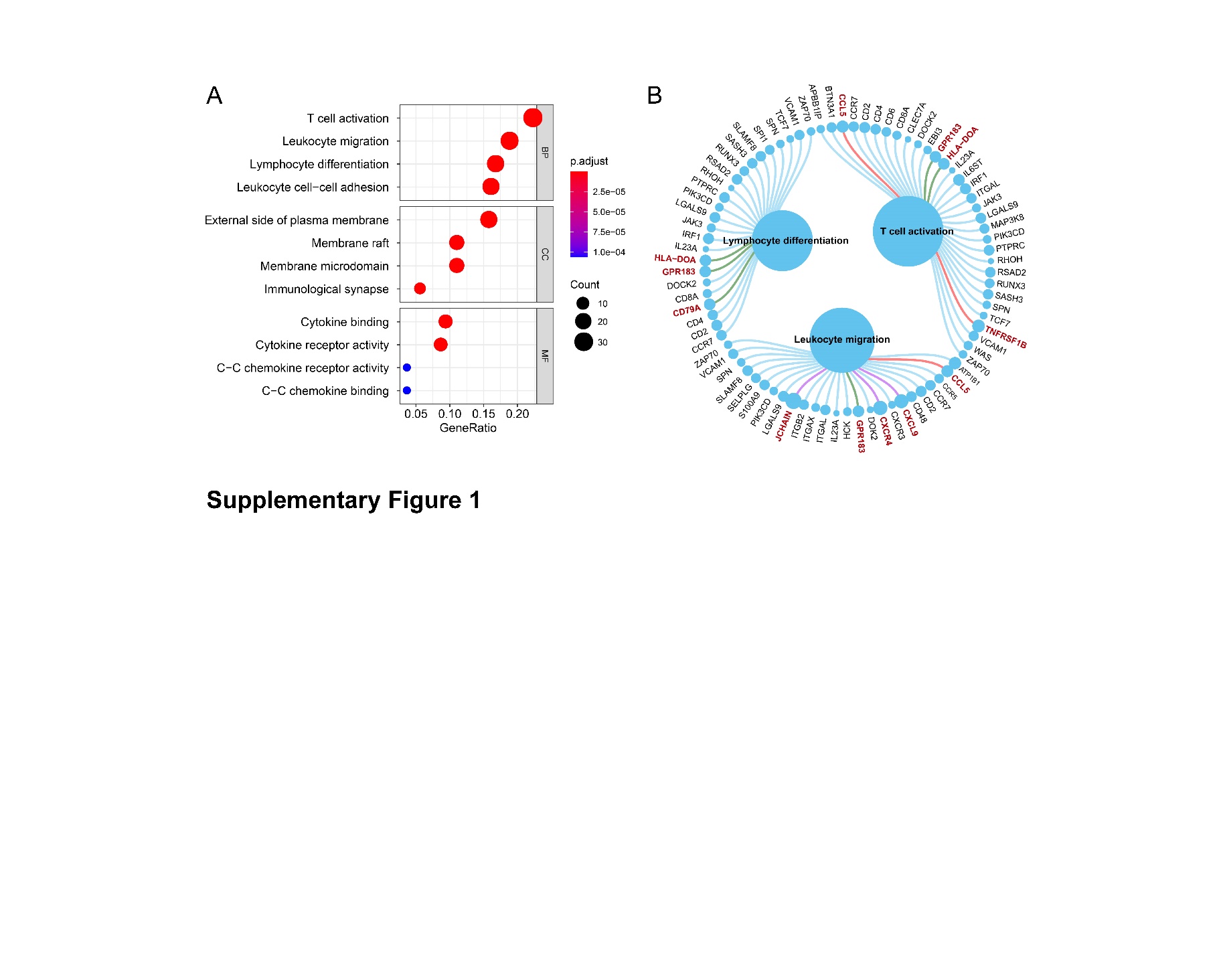
 **Supplementary Figure 1. Pathway enrichment of the top 150 exemplar genes.** (A) The function enrichment of the top 150 exemplar genes in Gene Ontology (GO) Biological Process (BP), Molecular Function (MF), and Cellular Component (CC). (B) The enriched genes in T cell activation, Leukocyte migration, and Lymphocyte differentiation pathways. The top-weighted genes in each pathway were marked with red color.


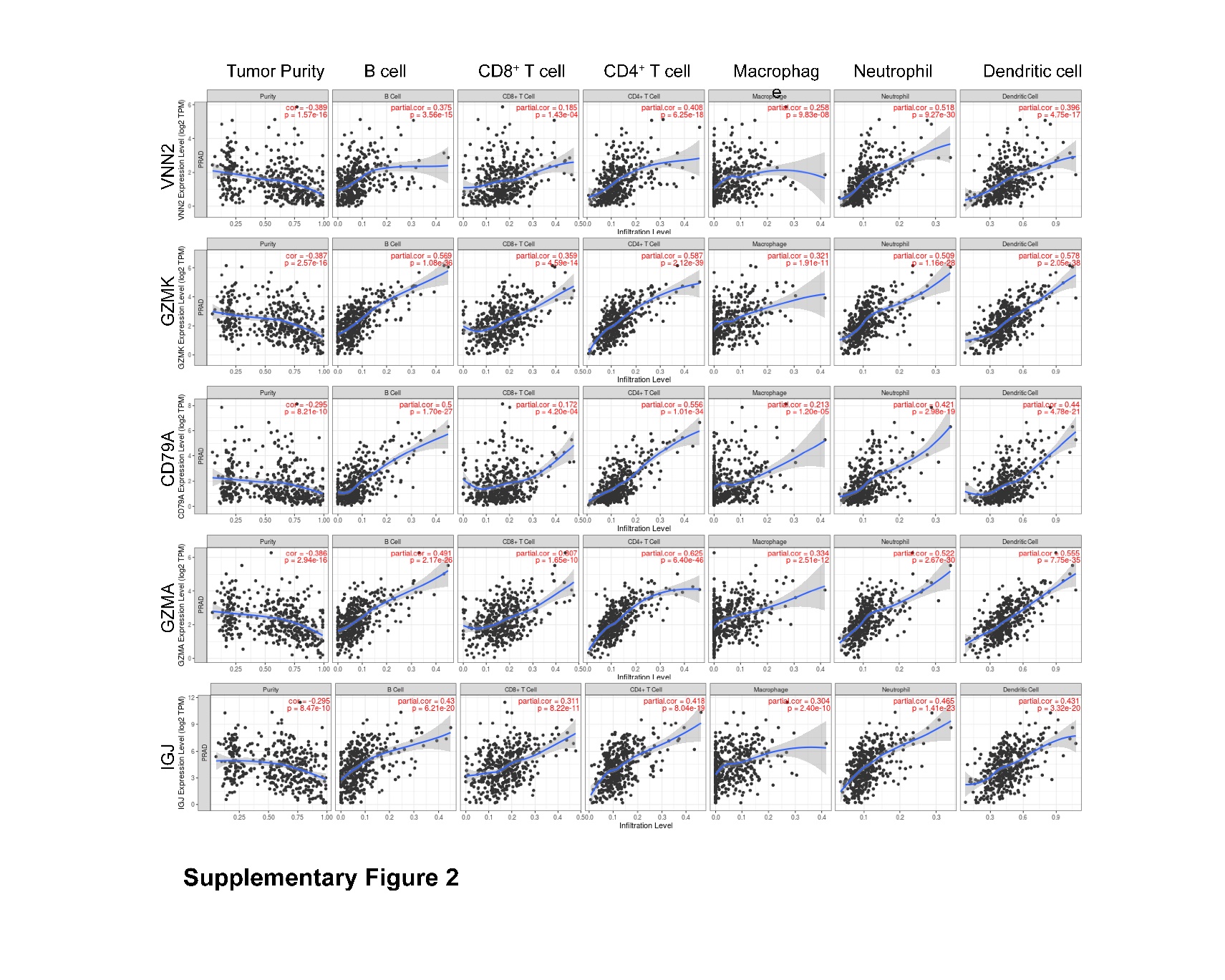


**Supplementary Figure 2. The association between the infiltration of immunocytes and the top 5 exemplar genes of immune factor.** The results obtained from TIMER (<https://cistrome.shinyapps.io/timer>), the abundances of six immunocytes (B cells, CD4+ T cells, CD8+ T cells, Neutrophils, Macrophages, and Dendritic cells) are estimated by TIMER algorithm, correlation displays by purity-corrected partial Spearman’s rho value.


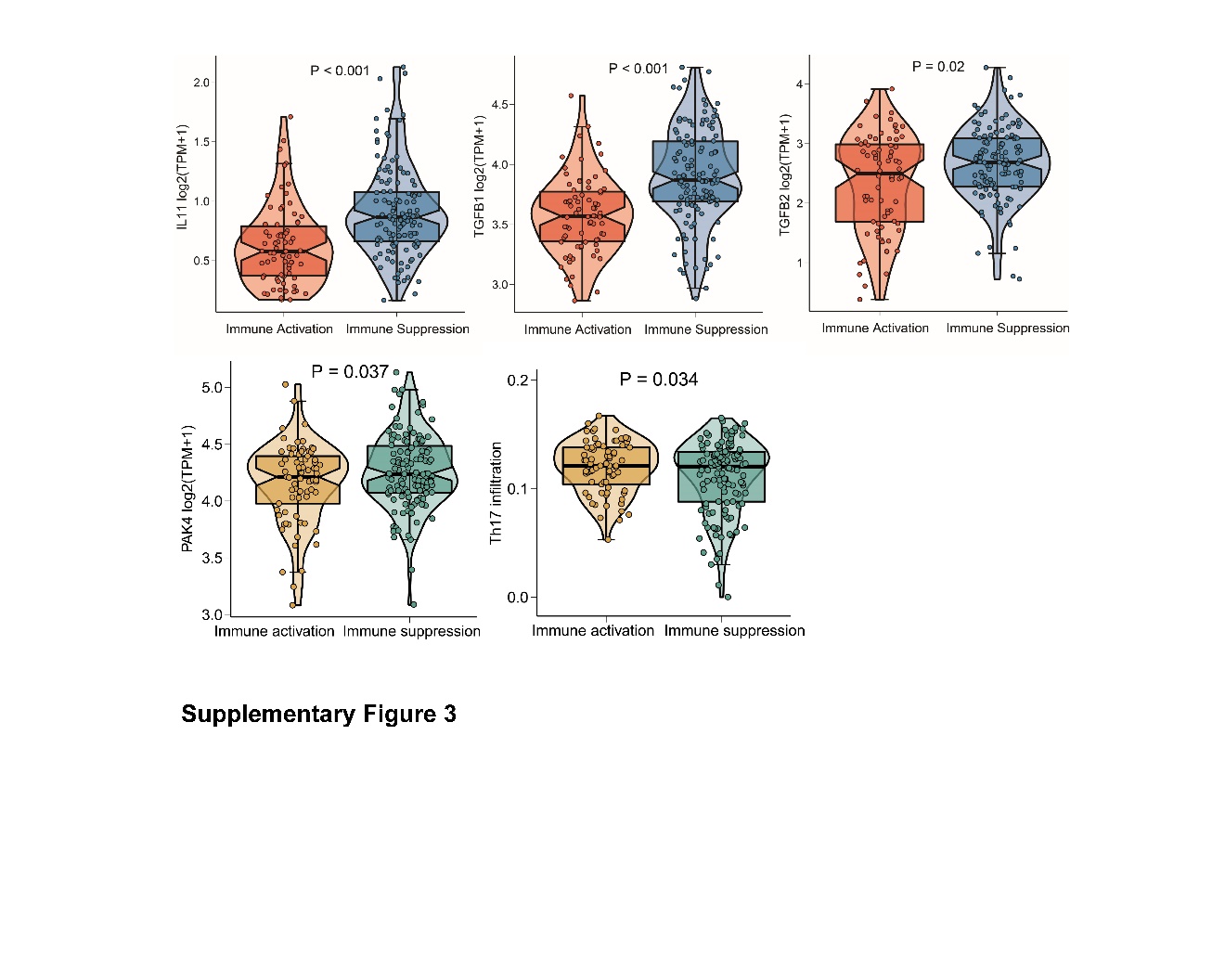


**Supplementary Figure 3. The different expressions of stromal markers and infiltration of Th17 cells in immune activated and suppressed classes.** The cooperation between two groups was conducted by Student T-test.


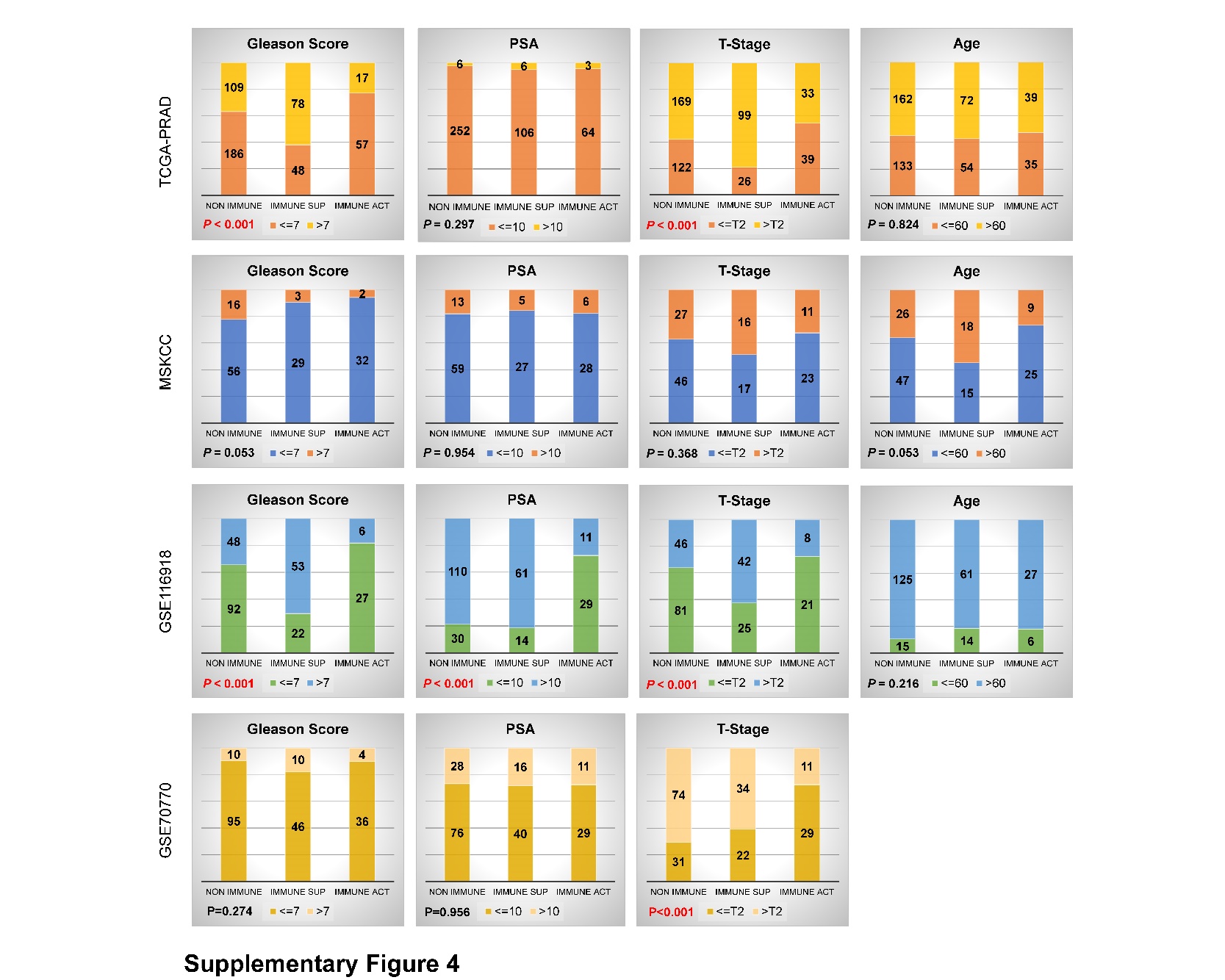


**Supplementary Figure 4. The distribution of Gleason score, PSA, Age and pathological T stage among three immunophenotypes in TCGA-PRAD, MSKCC, GSE116918 and GSE70770 cohorts.** The difference was conducted by Chi-square test.


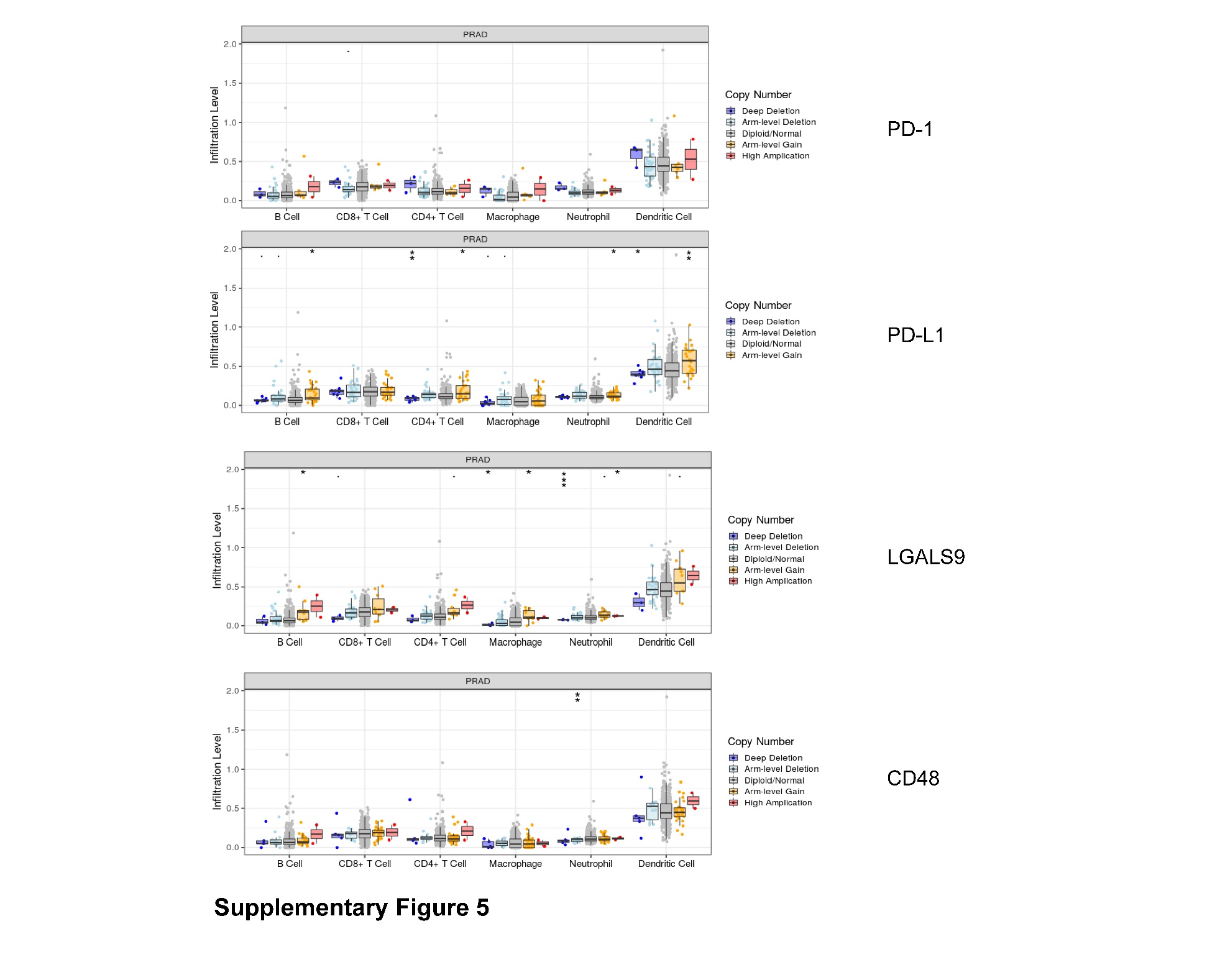


**Supplementary Figure 5. The association between copy number variation of immune checkpoints and immunocyte infiltration.** The infiltration level for each copy number category is compared with the normal using a two-sided Wilcoxon rank-sum test.


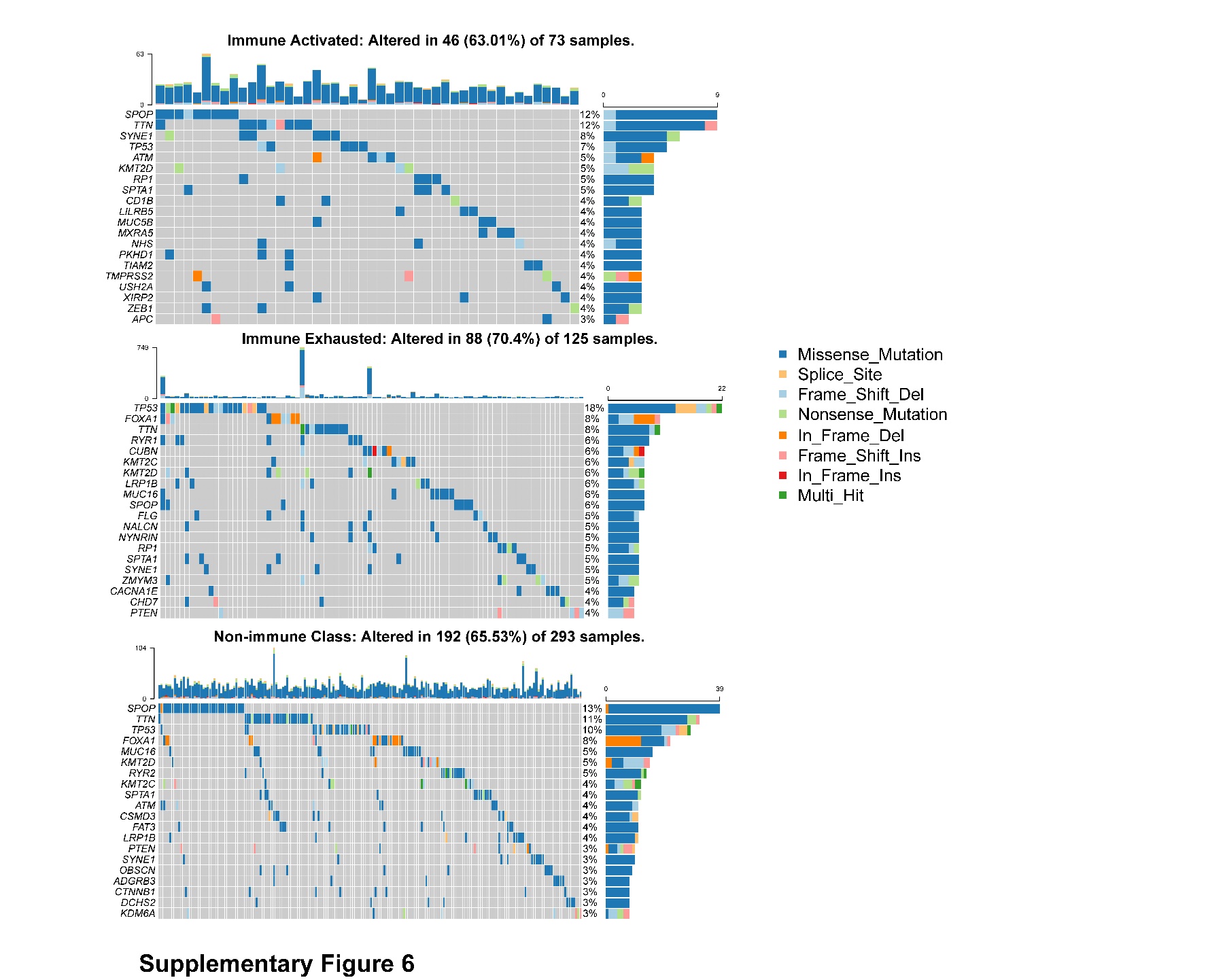


**Supplementary Figure 6. The mutational landscape showed the top mutated genes in non-immune, immune-activated, and immune-suppressed subgroups in TCGA-PRAD cohort.** TCGA-PRAD, The Cancer Genome Atlas-prostate adenocarcinoma.


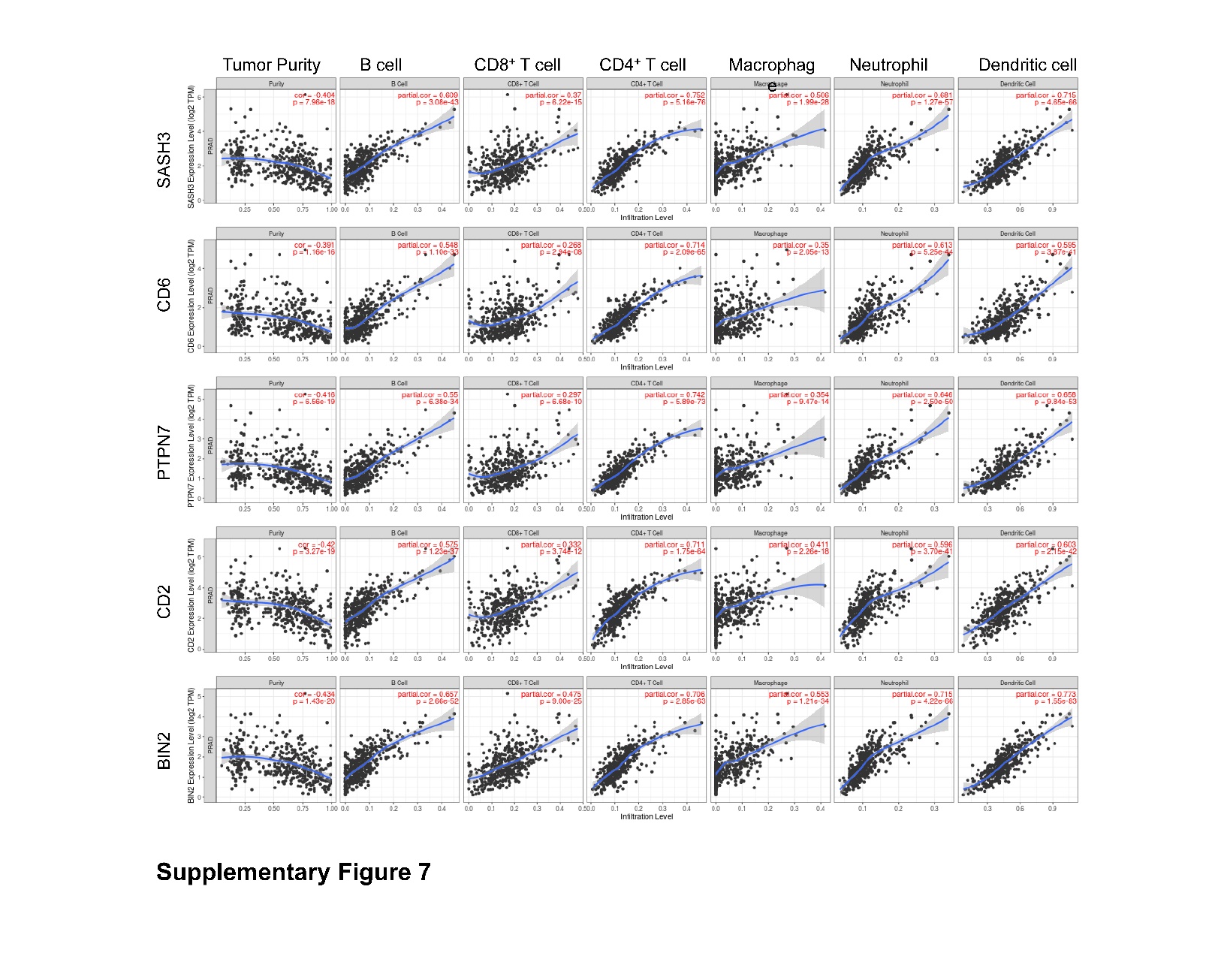


**Supplementary Figure 7. The association between the infiltration of immunocytes and the top 5 differentially expressed genes among immune and non-immune classes.** The results obtained from TIMER (<https://cistrome.shinyapps.io/timer>), the abundances of six immunocytes (B cells, CD4+ T cells, CD8+ T cells, Neutrophils, Macrophages, and Dendritic cells) are estimated by TIMER algorithm, correlation displayes by purity-corrected partial Spearman’s rho value.


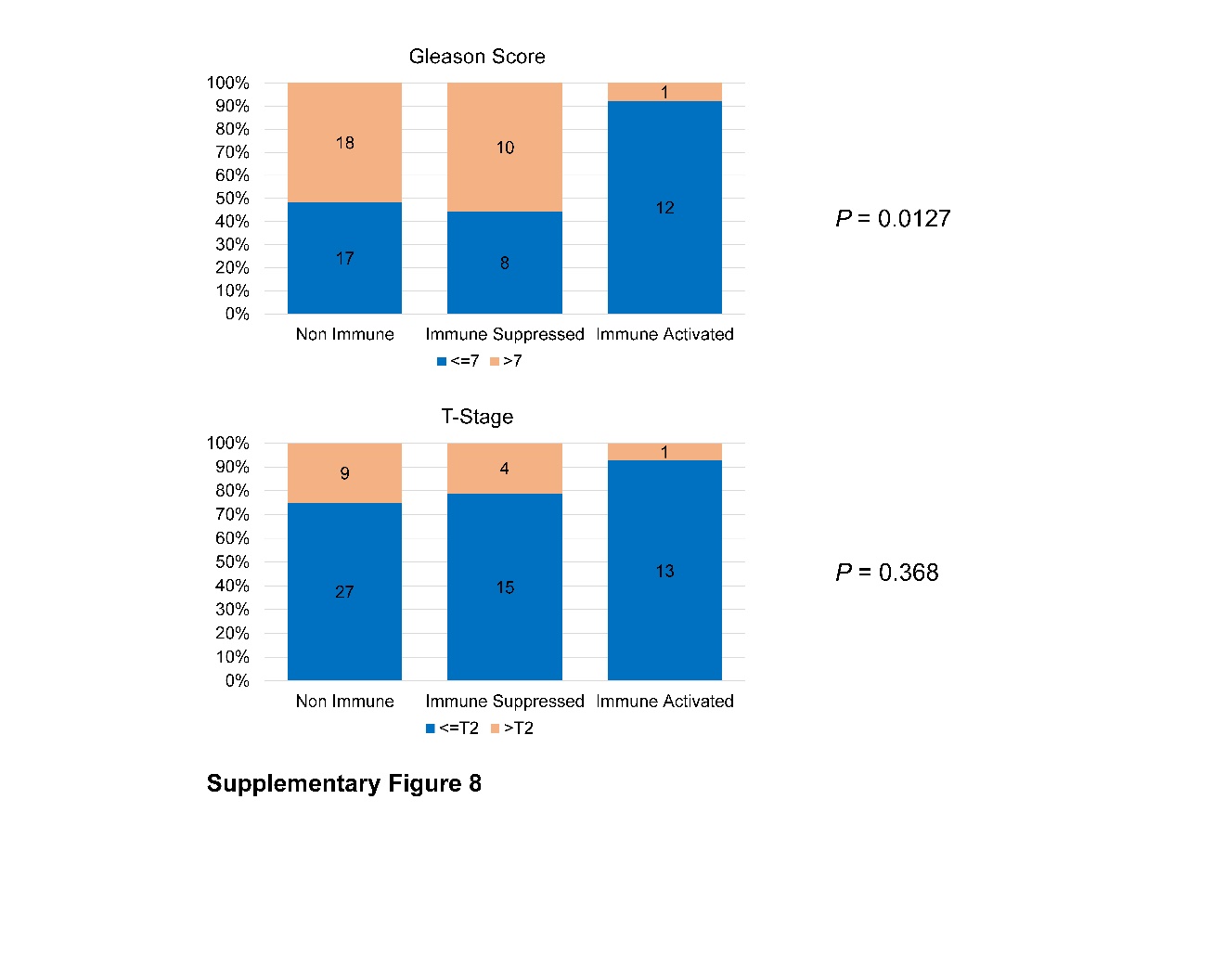


**Supplementary Figure 8. The distribution of clinicopathological features among three immunophenotypes in AHMU-PC cohort.** The difference between three subtypes was conducted by Chi-square test; Three samples lack of Gleason score data in in AHMU-PC cohort.


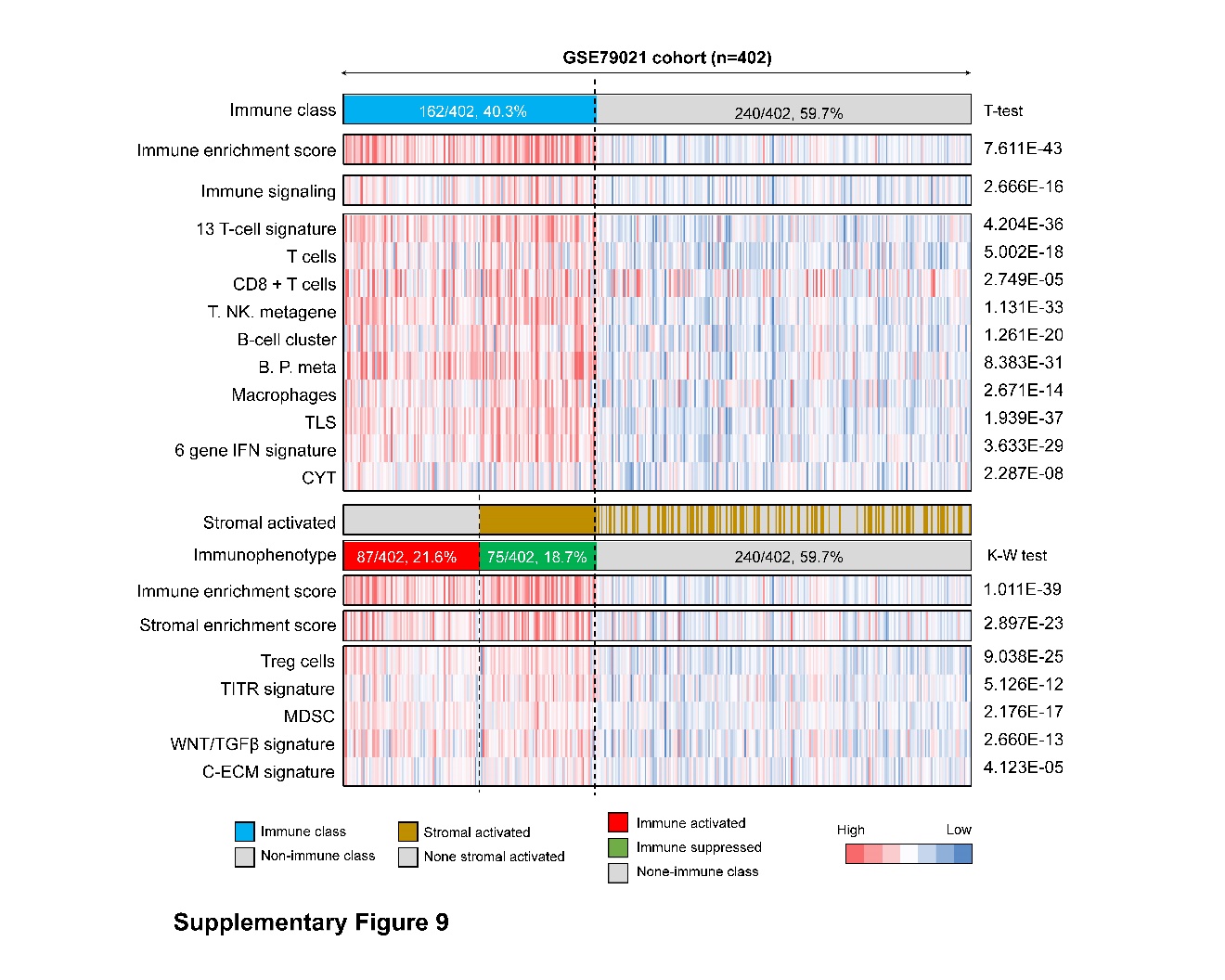


**Supplementary Figure 9. Successful validation of the immunophenotypes among the GSE79021 cohort.** Consensus-clustered heatmap by the top 150 differentially expressed genes among immune and non-immune classes in the training cohort were conducted to generate the immune and non-immune classes in the validation cohort, and further divided into suppressed and activation classes by activated stroma signature; In the heat map, high and low single-sample gene set enrichment scores are represented in red and blue, respectively. Positive prediction of activated stroma signature as per NTP is indicated in brown and its absence is in grey. CYT, cytolytic activity score; TITR, tumor-infiltrating Tregs; MDSC, myeloid-derived suppressor cell; TLS, tertiary lymphoid structure; C-ECM, cancer-associated extracellular matrix.


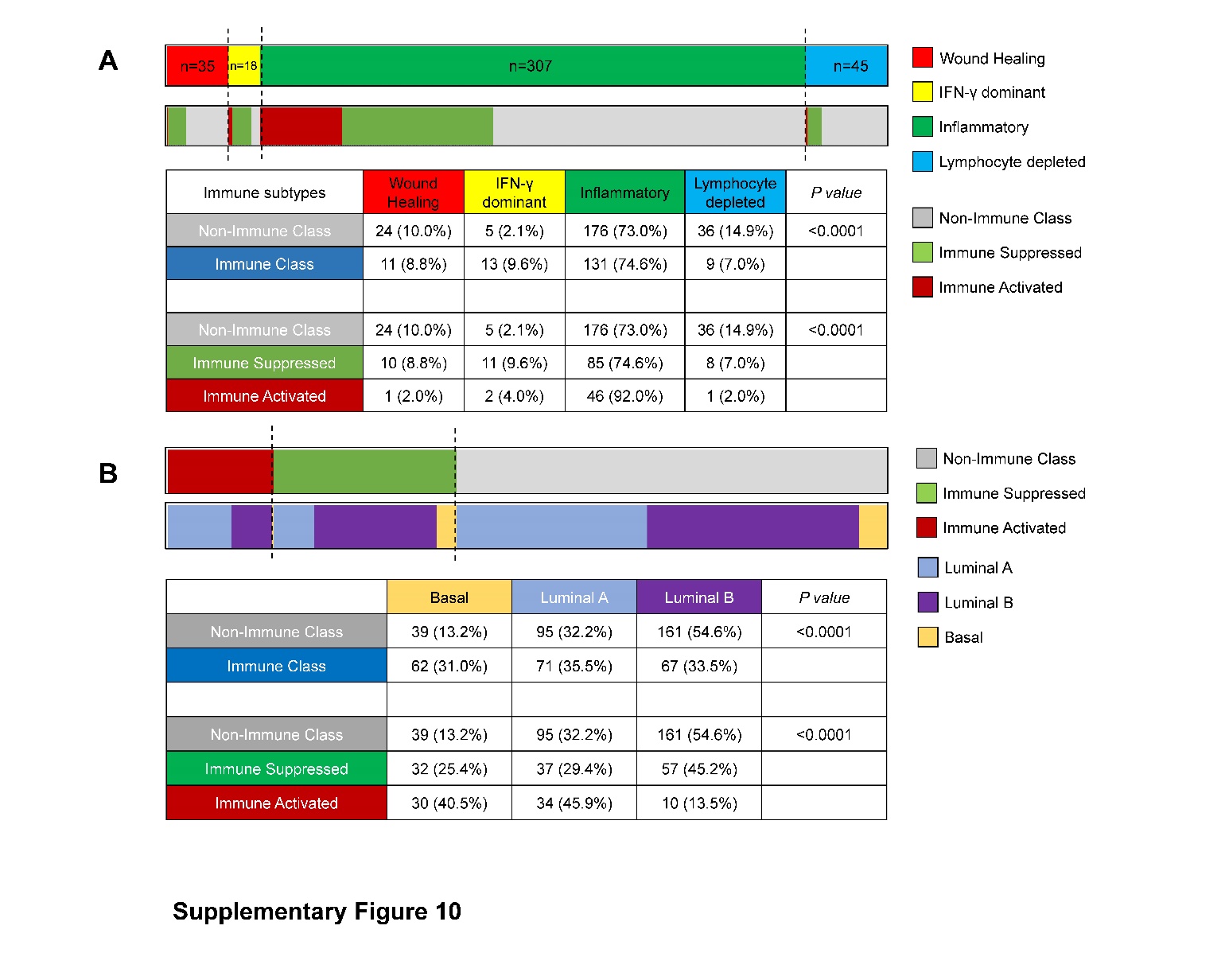


**Supplementary Figure 10. Association of the three immunophenotypes with the six pan-cancer immune molecular subgroups (A) and PAM50 molecular subtypes (B).** The difference was conducted by Chi-square test.


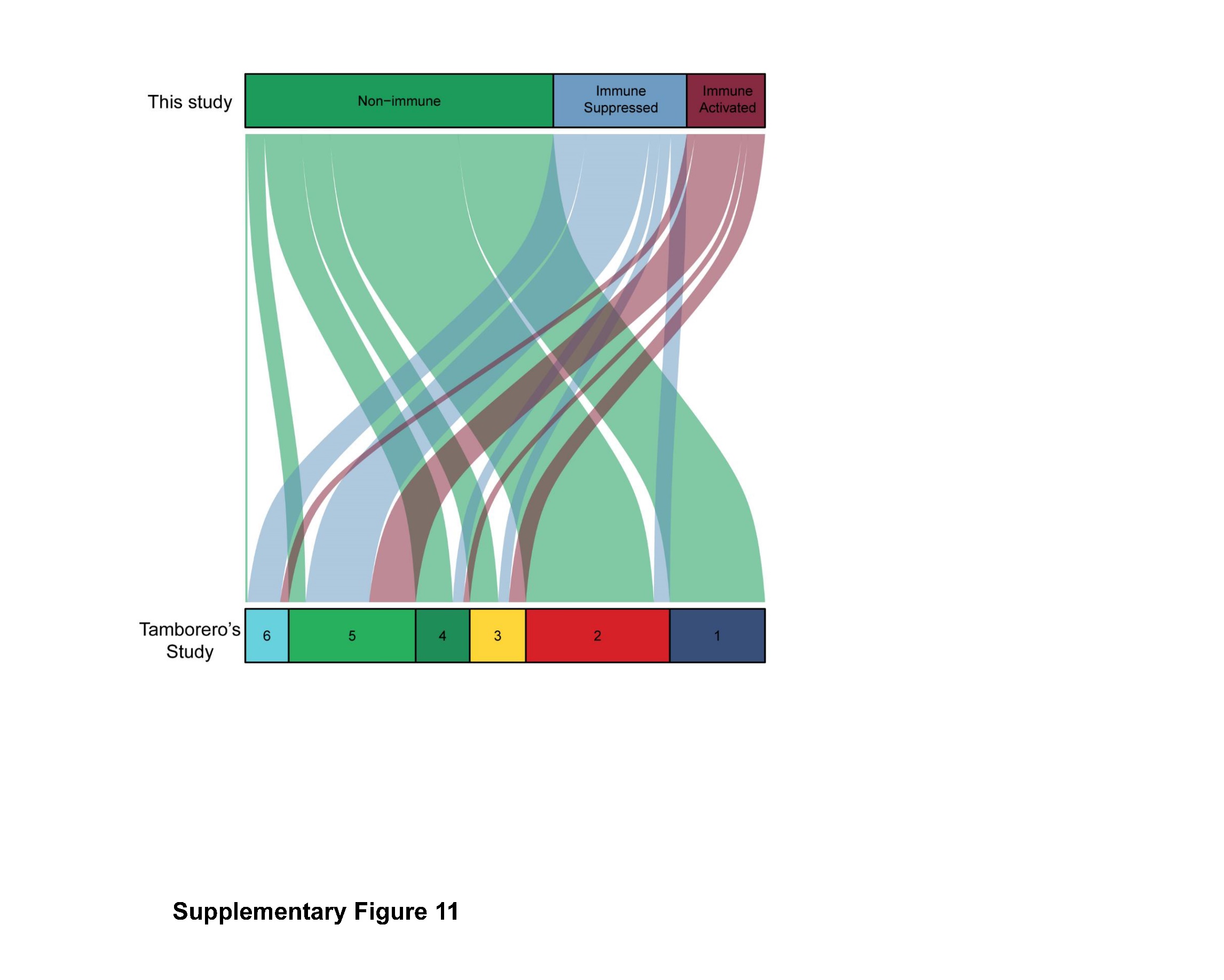


**Supplementary Figure 11. Association of the three immunophenotypes with the six molecular subgroups defined by Tamborero’s study displayed by Sankey plot.**
