# Supplementary material for: Immune response drives outcomes in prostate cancer: implications for immunotherapy
Source: Mol Oncol. 2020 Dec 29;15(5):1358–75. doi: 10.1002/1878-0261.12887 (PMC8096785; doi:10.1002/1878-0261.12887)
Supplement: Supplementary file 2 — Table S1. Immune associated gene signatures used in this study. Table S2. Top 150 weighted genes of factor 2. Table S3. Differentially mutated genes based on immune molecular subtypes. Table S4. Top 150 differentially expressed genes in immune class compare with non‐immune class in the TCGA training cohort. [file MOL2-15-1358-s002.docx]

# **Supplementary Table 1. Immune associated gene signatures used in this study**

| **Signature Name** | **Reference** |
| --- | --- |
| Immune enrichment score | Yoshihara *et al.* Nat Commun. 2013;4:2612 |
| Stromal enrichment score | Yoshihara *et al.* Nat Commun. 2013;4:2612 |
| Immune signalling molecules | Cancer Genome Atlas Network. Cell. 2015;161:1681-96 |
| 13 T-cell signature | Spranger *et al.* Proc Natl Acad Sci U S A. 2016;113(48):E7759-E7768. |
| T cells | Bindea *et al.* Immunity. 2013;39:782-95 |
| CD8 T cells | Bindea *et al.* Immunity. 2013;39:782-95 |
| Treg cells | Angelova *et al.* Genome Biol. 2015;16:64 |
| TITR signature | Magnuson *et al.* Proceedings of the National Academy of Sciences of the United States of America. 2018;115(45):E10672-e81 |
| MDSC | Angelova *et al.* Genome Biol. 2015;16:64 |
| T.NK. metagene | Alistar *et al.* Genome Med. 2014;6:80 |
| B-cell cluster | Iglesia *et al.* Clin Cancer Res. 2014;20(14):3818–3829. |
| B.P. metagene | Alistar *et al.* Genome Med. 2014;6:80 |
| Macrophages | Bindea *et al.* Immunity. 2013;39:782-95 |
| TLS | Finkin *et al.* Nat Immunol. 2015;16:1235-44 |
| 6-gene IFN signature | Chow *et al.* J Clin Oncol. 34, (suppl; abstr 6010) 2016 |
| CYT | Rooney *et al.* Cell. 2015;160:48-61 |
| WNT/TGF-β signature | Lachenmayer *et al.* Clin Cancer Res. 2012;18:4997-5007 |
| C-ECM signature | Chakravarthy *et al.* Nature Communications. 2018;9(1) |
| Six immune subtypes of Pan-Cancer Atlas | Thorsson *et al.* Immunity. 2018;5(5):489-500 |
| PAM50 pan-cancer | Zhao *et al.* Clin Cancer Res. 2019;25(8):2450-2457 |

Abbreviations: TITR, tumour-infiltrating Tregs; MDSC, myeloid-derived suppressor cell; IFN: interferon; TLS, tertiary lymphoid structure; CYT, cytolytic activity score; C-ECM, cancer-associated extracellular matrix.

# **Supplementary Table 2. Top 150 weighted genes of factor 2.**

| **Name** | **Weight in factor 2** | **Max weight**  **in other factors** | **Difference** |
| --- | --- | --- | --- |
| VNN2 | 1.319 | 0.371 | 0.948 |
| GZMK | 1.133 | 0.467 | 0.666 |
| CD79A | 1.241 | 0.612 | 0.629 |
| GZMA | 1.201 | 0.581 | 0.62 |
| IGJ | 2.004 | 1.396 | 0.608 |
| CCR7 | 1.106 | 0.517 | 0.589 |
| CD247 | 0.852 | 0.277 | 0.575 |
| SLAMF7 | 1.059 | 0.486 | 0.573 |
| RSAD2 | 1.105 | 0.533 | 0.572 |
| BIN2 | 0.977 | 0.406 | 0.571 |
| CD48 | 1.137 | 0.586 | 0.551 |
| CD53 | 1.407 | 0.861 | 0.546 |
| CYTIP | 1.153 | 0.613 | 0.54 |
| IL10RA | 1.184 | 0.652 | 0.532 |
| SLAMF8 | 1.112 | 0.589 | 0.523 |
| HCLS1 | 1.199 | 0.685 | 0.514 |
| IL18BP | 1.038 | 0.526 | 0.512 |
| JAK3 | 0.932 | 0.431 | 0.501 |
| RGS16 | 1.006 | 0.51 | 0.496 |
| CCR5 | 0.993 | 0.499 | 0.494 |
| PIM2 | 1.061 | 0.574 | 0.487 |
| SKAP1 | 0.883 | 0.397 | 0.486 |
| CYTH4 | 0.878 | 0.393 | 0.485 |
| CD37 | 1.076 | 0.592 | 0.484 |
| IL4I1 | 0.838 | 0.362 | 0.476 |
| PLEK | 1.197 | 0.722 | 0.475 |
| CD6 | 0.995 | 0.525 | 0.47 |
| HLA-DRA | 2.24 | 1.775 | 0.465 |
| BIRC3 | 1.307 | 0.855 | 0.452 |
| CXCR4 | 1.655 | 1.212 | 0.443 |
| CP | 1.301 | 0.858 | 0.443 |
| RUNX3 | 1.013 | 0.571 | 0.442 |
| TNFAIP2 | 1.534 | 1.099 | 0.435 |
| HLA-DOA | 1.247 | 0.816 | 0.431 |
| MAP3K8 | 1.046 | 0.619 | 0.427 |
| IFIT1 | 1.045 | 0.623 | 0.422 |
| SASH3 | 1.09 | 0.669 | 0.421 |
| MX2 | 0.704 | 0.286 | 0.418 |
| CSF2RB | 0.956 | 0.538 | 0.418 |
| IFI44 | 1.239 | 0.831 | 0.408 |
| ZAP70 | 0.844 | 0.445 | 0.399 |
| GEM | 1.355 | 0.957 | 0.398 |
| PCDH17 | 0.844 | 0.449 | 0.395 |
| ITGAL | 1.085 | 0.695 | 0.39 |
| SPOCK2 | 1.142 | 0.754 | 0.388 |
| CD2 | 1.168 | 0.783 | 0.385 |
| IGSF6 | 0.867 | 0.483 | 0.384 |
| ITGAX | 0.874 | 0.494 | 0.38 |
| PLCB2 | 0.796 | 0.42 | 0.376 |
| LGALS9 | 1.131 | 0.758 | 0.373 |
| CASP1 | 0.983 | 0.613 | 0.37 |
| TAP1 | 1.297 | 0.927 | 0.37 |
| EBI3 | 0.846 | 0.478 | 0.368 |
| WAS | 0.956 | 0.591 | 0.365 |
| EVI2B | 1.142 | 0.78 | 0.362 |
| ETV7 | 0.796 | 0.436 | 0.36 |
| APBB1IP | 0.879 | 0.528 | 0.351 |
| ARHGAP25 | 0.855 | 0.505 | 0.35 |
| GPR183 | 1.237 | 0.895 | 0.342 |
| HLA-DQA1 | 1.222 | 0.885 | 0.337 |
| RGS1 | 1.373 | 1.037 | 0.336 |
| MAP4K1 | 0.837 | 0.503 | 0.334 |
| NR4A2 | 1.227 | 0.895 | 0.332 |
| SF3B1 | 1.308 | 0.976 | 0.332 |
| SELPLG | 1.03 | 0.7 | 0.33 |
| STAB1 | 0.976 | 0.646 | 0.33 |
| TNFRSF1B | 1.407 | 1.081 | 0.326 |
| IRF8 | 1.037 | 0.711 | 0.326 |
| SPI1 | 1.013 | 0.688 | 0.325 |
| CD69 | 1.133 | 0.811 | 0.322 |
| CIITA | 0.805 | 0.485 | 0.32 |
| SPN | 0.843 | 0.523 | 0.32 |
| IFIT2 | 0.933 | 0.615 | 0.318 |
| LAIR1 | 0.76 | 0.442 | 0.318 |
| CST7 | 1.025 | 0.707 | 0.318 |
| KLF5 | 1.328 | 1.011 | 0.317 |
| SLCO2B1 | 0.932 | 0.616 | 0.316 |
| BTN3A1 | 1.069 | 0.754 | 0.315 |
| HCK | 0.904 | 0.596 | 0.308 |
| LCP2 | 0.911 | 0.606 | 0.305 |
| IRF1 | 1.165 | 0.862 | 0.303 |
| ROBO1 | 1.133 | 0.831 | 0.302 |
| PLXNC1 | 0.796 | 0.496 | 0.3 |
| CXCL9 | 1.609 | 1.309 | 0.3 |
| CD4 | 1.098 | 0.799 | 0.299 |
| ITGB2 | 1.071 | 0.777 | 0.294 |
| ACHE | 0.796 | 0.503 | 0.293 |
| MAP3K1 | 0.887 | 0.595 | 0.292 |
| NPL | 0.727 | 0.435 | 0.292 |
| CD8A | 0.934 | 0.644 | 0.29 |
| SAMSN1 | 0.783 | 0.495 | 0.288 |
| VCAM1 | 1.062 | 0.774 | 0.288 |
| CXCR6 | 0.726 | 0.441 | 0.285 |
| CLEC7A | 0.695 | 0.415 | 0.28 |
| PPP1R16B | 0.822 | 0.542 | 0.28 |
| MS4A6A | 0.896 | 0.617 | 0.279 |
| TCF7 | 0.786 | 0.512 | 0.274 |
| ADORA2B | 0.639 | 0.368 | 0.271 |
| ATP1B1 | 1.36 | 1.09 | 0.27 |
| CTSS | 1.206 | 0.936 | 0.27 |
| AMIGO2 | 1.01 | 0.741 | 0.269 |
| IFIT3 | 1.055 | 0.787 | 0.268 |
| DOCK2 | 0.793 | 0.526 | 0.267 |
| IL6ST | 1.246 | 0.98 | 0.266 |
| SRGN | 1.468 | 1.204 | 0.264 |
| TGFBI | 1.11 | 0.847 | 0.263 |
| MMP2 | 1.387 | 1.126 | 0.261 |
| DOK2 | 0.716 | 0.457 | 0.259 |
| DYRK3 | 0.599 | 0.342 | 0.257 |
| CCL5 | 1.307 | 1.05 | 0.257 |
| FPR1 | 0.766 | 0.512 | 0.254 |
| PTPN7 | 0.604 | 0.351 | 0.253 |
| S100A9 | 1.084 | 0.832 | 0.252 |
| STX11 | 0.714 | 0.463 | 0.251 |
| HLA-F | 1.104 | 0.858 | 0.246 |
| SEMA7A | 0.653 | 0.408 | 0.245 |
| FAIM3 | 0.954 | 0.709 | 0.245 |
| S1PR4 | 0.674 | 0.433 | 0.241 |
| PTPRC | 1.115 | 0.874 | 0.241 |
| IL23A | 0.511 | 0.271 | 0.24 |
| IL2RB | 0.906 | 0.667 | 0.239 |
| GNG12 | 1.246 | 1.008 | 0.238 |
| EGLN3 | 0.695 | 0.457 | 0.238 |
| CXCR3 | 0.79 | 0.561 | 0.229 |
| SAMD9 | 0.814 | 0.585 | 0.229 |
| SMEK1 | 0.946 | 0.717 | 0.229 |
| FCGR2A | 0.743 | 0.517 | 0.226 |
| SCUBE2 | 1.385 | 1.159 | 0.226 |
| KCNAB2 | 0.82 | 0.596 | 0.224 |
| CTSC | 0.945 | 0.721 | 0.224 |
| FPR3 | 0.942 | 0.72 | 0.222 |
| SEC61A2 | 0.602 | 0.383 | 0.219 |
| IL2RG | 1.138 | 0.924 | 0.214 |
| IL32 | 1.15 | 0.936 | 0.214 |
| GZMB | 0.641 | 0.428 | 0.213 |
| FOLR2 | 0.952 | 0.739 | 0.213 |
| OLFML2B | 0.905 | 0.693 | 0.212 |
| PFKFB3 | 1.06 | 0.849 | 0.211 |
| HMGB2 | 1.097 | 0.886 | 0.211 |
| PIK3CD | 0.894 | 0.684 | 0.21 |
| RHOH | 0.672 | 0.463 | 0.209 |
| PTAFR | 0.772 | 0.566 | 0.206 |
| KCNJ2 | 0.624 | 0.419 | 0.205 |
| TNFSF15 | 0.835 | 0.63 | 0.205 |
| NUAK1 | 1.006 | 0.802 | 0.204 |
| EPB41L2 | 1.067 | 0.864 | 0.203 |
| RNF19A | 1.025 | 0.822 | 0.203 |
| OLR1 | 0.808 | 0.606 | 0.202 |
| CALML4 | 0.499 | 0.297 | 0.202 |
| SOCS3 | 1.401 | 1.199 | 0.202 |

# **Supplementary Table 3. Differentially mutated genes based on immune molecular subtypes.**

| **Mutated genes** | **Immune Activation Subtype**  **N=74** | **Immune Suppression Subtype**  **N=126** | **Non-Immune Class**  **N=295** | ***P value*** |
| --- | --- | --- | --- | --- |
| TP53 | 4 | 24 | 29 | <0.001 |
| SPOP | 10 | 7 | 39 | <0.001 |
| BRCA2 | 2 | 6 | 1 | 0.029 |
| SCN10A | 2 | 5 | 2 | 0.031 |
| C14orf115 | 0 | 6 | 1 | 0.038 |
| OR4C6 | 3 | 3 | 1 | 0.038 |
| KAL1 | 0 | 4 | 1 | 0.04 |
| CHRNA6 | 0 | 4 | 0 | 0.005 |
| KIAA1012 | 0 | 4 | 0 | 0.007 |
| OR10R2 | 2 | 2 | 0 | 0.009 |
| PLIN3 | 0 | 3 | 0 | 0.01 |
| MOSPD3 | 0 | 3 | 0 | 0.024 |
| GMPR | 0 | 3 | 0 | 0.037 |
| DPPA5 | 0 | 3 | 0 | 0.041 |
| C10orf99 | 2 | 0 | 0 | 0.027 |

# **Supplementary Table 4. Top 150 differentially expressed genes in immune class compare with non-immune class in the TCGA training cohort.**

| **Gene** | **logFC** | **AveExpr** | **t** | **P.Value** | **adj.P.Val** | **B** |
| --- | --- | --- | --- | --- | --- | --- |
| SASH3 | 1.062645 | 2.053972 | 27.60796 | 1.17E-102 | 1.76E-100 | 223.6671 |
| CD6 | 0.896031 | 1.148307 | 26.58106 | 9.47E-98 | 7.10E-96 | 212.3968 |
| PTPN7 | 0.798828 | 1.054899 | 26.0794 | 2.43E-95 | 1.21E-93 | 206.8627 |
| CD2 | 1.206715 | 2.38856 | 25.87283 | 2.40E-94 | 8.99E-93 | 204.579 |
| BIN2 | 0.830798 | 1.330384 | 25.68265 | 1.98E-93 | 5.93E-92 | 202.4742 |
| CD48 | 1.061612 | 1.913432 | 25.57875 | 6.27E-93 | 1.57E-91 | 201.3232 |
| ITGAL | 1.009295 | 1.607309 | 25.35181 | 7.80E-92 | 1.67E-90 | 198.8073 |
| CD247 | 0.826952 | 1.066891 | 25.25031 | 2.41E-91 | 4.52E-90 | 197.681 |
| MAP4K1 | 0.825714 | 1.38623 | 25.21653 | 3.51E-91 | 5.86E-90 | 197.3061 |
| WAS | 0.809329 | 2.112 | 25.18542 | 4.97E-91 | 7.45E-90 | 196.9608 |
| IL10RA | 0.951437 | 1.778299 | 25.12716 | 9.50E-91 | 1.30E-89 | 196.3138 |
| CYTH4 | 0.757889 | 1.382842 | 24.73669 | 7.36E-89 | 9.20E-88 | 191.9738 |
| SELPLG | 0.966485 | 2.421796 | 24.47334 | 1.39E-87 | 1.60E-86 | 189.0426 |
| CCR5 | 0.914448 | 1.379335 | 24.39403 | 3.37E-87 | 3.44E-86 | 188.1593 |
| SLAMF7 | 0.984142 | 1.260339 | 24.39221 | 3.44E-87 | 3.44E-86 | 188.1391 |
| CXCR3 | 0.875182 | 1.102048 | 24.33501 | 6.51E-87 | 6.10E-86 | 187.5018 |
| DOK2 | 0.846534 | 1.629889 | 24.01598 | 2.30E-85 | 2.03E-84 | 183.9453 |
| APBB1IP | 0.833045 | 1.504287 | 23.98528 | 3.24E-85 | 2.70E-84 | 183.603 |
| CD4 | 1.013571 | 2.632109 | 23.81067 | 2.29E-84 | 1.80E-83 | 181.6548 |
| HCLS1 | 0.868794 | 2.513285 | 23.66046 | 1.23E-83 | 9.21E-83 | 179.9781 |
| JAK3 | 0.830808 | 1.407756 | 23.44342 | 1.39E-82 | 9.95E-82 | 177.5544 |
| SPN | 0.806773 | 1.182018 | 23.36934 | 3.19E-82 | 2.18E-81 | 176.7268 |
| CD53 | 1.005084 | 3.051327 | 23.07951 | 8.21E-81 | 5.35E-80 | 173.4882 |
| GZMA | 1.066366 | 2.112556 | 22.79872 | 1.91E-79 | 1.17E-78 | 170.3491 |
| SPI1 | 0.824655 | 2.628564 | 22.79676 | 1.95E-79 | 1.17E-78 | 170.3272 |
| PLEK | 1.020789 | 2.008867 | 22.64047 | 1.13E-78 | 6.49E-78 | 168.5796 |
| ITGB2 | 0.941223 | 2.699275 | 22.61939 | 1.42E-78 | 7.92E-78 | 168.3439 |
| IL2RG | 1.085483 | 2.55158 | 22.33812 | 3.34E-77 | 1.79E-76 | 165.1985 |
| IL2RB | 1.024225 | 1.636903 | 22.18115 | 1.94E-76 | 1.00E-75 | 163.4429 |
| CST7 | 1.00771 | 2.177481 | 22.16146 | 2.42E-76 | 1.21E-75 | 163.2228 |
| CYTIP | 0.967192 | 1.592752 | 22.13198 | 3.36E-76 | 1.63E-75 | 162.8931 |
| CCR7 | 1.095795 | 1.276432 | 22.12213 | 3.76E-76 | 1.76E-75 | 162.783 |
| HCK | 0.849179 | 1.97024 | 22.04507 | 8.91E-76 | 4.05E-75 | 161.9212 |
| CD37 | 0.942431 | 2.043382 | 21.96633 | 2.15E-75 | 9.50E-75 | 161.0408 |
| RUNX3 | 0.869273 | 1.564123 | 21.84485 | 8.41E-75 | 3.60E-74 | 159.6824 |
| PIK3CD | 0.791696 | 1.653901 | 21.70883 | 3.86E-74 | 1.61E-73 | 158.1617 |
| CIITA | 0.778446 | 1.248782 | 21.67031 | 5.95E-74 | 2.41E-73 | 157.7311 |
| GZMK | 1.057318 | 1.537541 | 21.64345 | 8.03E-74 | 3.17E-73 | 157.4308 |
| S1PR4 | 0.791904 | 1.140545 | 21.5719 | 1.79E-73 | 6.89E-73 | 156.6311 |
| EVI2B | 1.007576 | 2.172317 | 21.50749 | 3.69E-73 | 1.38E-72 | 155.9112 |
| SLAMF8 | 0.861649 | 1.635616 | 21.40801 | 1.12E-72 | 4.11E-72 | 154.7996 |
| CXCR6 | 0.750787 | 1.065168 | 21.37482 | 1.63E-72 | 5.82E-72 | 154.4288 |
| PLCB2 | 0.64902 | 1.450634 | 21.23067 | 8.19E-72 | 2.86E-71 | 152.8184 |
| TNFRSF1B | 0.830166 | 2.926368 | 21.22015 | 9.21E-72 | 3.14E-71 | 152.701 |
| LAIR1 | 0.682042 | 1.470947 | 21.07617 | 4.62E-71 | 1.54E-70 | 151.0931 |
| DOCK2 | 0.76318 | 1.082248 | 21.02386 | 8.29E-71 | 2.70E-70 | 150.5091 |
| HLA-DQA1 | 1.050888 | 2.981624 | 20.99443 | 1.15E-70 | 3.68E-70 | 150.1806 |
| LCP2 | 0.801073 | 1.690293 | 20.91389 | 2.84E-70 | 8.87E-70 | 149.2817 |
| PTAFR | 0.724667 | 1.457374 | 20.90239 | 3.23E-70 | 9.88E-70 | 149.1533 |
| ZAP70 | 0.789092 | 1.049081 | 20.82868 | 7.36E-70 | 2.21E-69 | 148.3309 |
| HLA-DOA | 0.969246 | 2.631143 | 20.82623 | 7.57E-70 | 2.23E-69 | 148.3036 |
| CCL5 | 1.123353 | 3.382455 | 20.73619 | 2.07E-69 | 5.98E-69 | 147.2992 |
| PTPRC | 1.153447 | 1.948297 | 20.64687 | 5.62E-69 | 1.59E-68 | 146.3032 |
| EBI3 | 0.766035 | 1.522473 | 20.54363 | 1.78E-68 | 4.95E-68 | 145.1524 |
| FAIM3 | 0.934249 | 1.907069 | 20.43089 | 6.28E-68 | 1.71E-67 | 143.8962 |
| ARHGAP25 | 0.696137 | 1.523525 | 20.4224 | 6.91E-68 | 1.85E-67 | 143.8016 |
| LGALS9 | 0.771918 | 2.146444 | 20.35233 | 1.51E-67 | 3.97E-67 | 143.0212 |
| CASP1 | 0.801788 | 1.844636 | 20.30728 | 2.50E-67 | 6.46E-67 | 142.5196 |
| CTSS | 1.006203 | 3.164626 | 20.22304 | 6.39E-67 | 1.63E-66 | 141.5819 |
| IL4I1 | 0.791099 | 1.337966 | 20.1967 | 8.58E-67 | 2.14E-66 | 141.2888 |
| HLA-DRA | 1.005307 | 6.482121 | 20.18709 | 9.55E-67 | 2.35E-66 | 141.1819 |
| MS4A6A | 0.805397 | 2.218844 | 20.11809 | 2.06E-66 | 4.99E-66 | 140.4143 |
| CD8A | 0.901992 | 1.767263 | 20.03935 | 4.96E-66 | 1.18E-65 | 139.5387 |
| IL18BP | 0.655112 | 2.188067 | 19.99968 | 7.72E-66 | 1.81E-65 | 139.0976 |
| SAMSN1 | 0.784333 | 1.359527 | 19.7624 | 1.09E-64 | 2.50E-64 | 136.4618 |
| CTSC | 0.728095 | 2.10241 | 19.15678 | 9.06E-62 | 2.06E-61 | 129.7528 |
| SKAP1 | 0.707509 | 1.274075 | 19.06567 | 2.49E-61 | 5.57E-61 | 128.7461 |
| CXCR4 | 1.027466 | 3.833323 | 18.98877 | 5.83E-61 | 1.29E-60 | 127.897 |
| IGSF6 | 0.727723 | 1.767834 | 18.89532 | 1.64E-60 | 3.56E-60 | 126.8659 |
| CSF2RB | 0.848534 | 1.368564 | 18.73081 | 1.01E-59 | 2.16E-59 | 125.0529 |
| ITGAX | 0.717795 | 1.630867 | 18.21793 | 2.87E-57 | 6.07E-57 | 119.4188 |
| HLA-F | 0.84295 | 3.291533 | 17.98892 | 3.55E-56 | 7.39E-56 | 116.9132 |
| KCNAB2 | 0.469855 | 1.64504 | 17.89749 | 9.66E-56 | 1.98E-55 | 115.9146 |
| CLEC7A | 0.641975 | 1.120053 | 17.79514 | 2.96E-55 | 6.00E-55 | 114.7982 |
| CD79A | 1.265215 | 1.604442 | 17.51496 | 6.30E-54 | 1.26E-53 | 111.7495 |
| IL32 | 0.851666 | 2.876667 | 17.5132 | 6.43E-54 | 1.27E-53 | 111.7304 |
| CD69 | 0.979326 | 1.711345 | 17.2413 | 1.24E-52 | 2.41E-52 | 108.7829 |
| IRF8 | 0.864378 | 1.730911 | 16.82362 | 1.13E-50 | 2.18E-50 | 104.2784 |
| SLCO2B1 | 0.723579 | 1.891455 | 16.70191 | 4.21E-50 | 8.00E-50 | 102.9714 |
| BIRC3 | 0.884621 | 2.356495 | 16.29434 | 3.34E-48 | 6.25E-48 | 98.61497 |
| SEMA7A | 0.489427 | 1.118305 | 16.22739 | 6.82E-48 | 1.26E-47 | 97.9024 |
| GPR183 | 0.97446 | 2.433596 | 16.22621 | 6.91E-48 | 1.26E-47 | 97.88983 |
| PPP1R16B | 0.735265 | 1.076339 | 16.19534 | 9.60E-48 | 1.74E-47 | 97.56161 |
| STAB1 | 0.652556 | 2.271453 | 16.16885 | 1.27E-47 | 2.27E-47 | 97.2801 |
| PLXNC1 | 0.634072 | 1.305913 | 16.03913 | 5.07E-47 | 8.95E-47 | 95.90371 |
| FOLR2 | 0.772608 | 2.463543 | 15.92386 | 1.73E-46 | 3.01E-46 | 94.68363 |
| ETV7 | 0.69874 | 1.550459 | 15.85064 | 3.75E-46 | 6.47E-46 | 93.91011 |
| GZMB | 0.744445 | 1.238519 | 15.75749 | 1.01E-45 | 1.71E-45 | 92.92775 |
| FPR3 | 0.795345 | 1.905202 | 15.68409 | 2.18E-45 | 3.68E-45 | 92.15502 |
| NPL | 0.525539 | 1.327497 | 15.40398 | 4.17E-44 | 6.95E-44 | 89.21764 |
| TNFAIP2 | 0.956357 | 3.280592 | 15.26064 | 1.87E-43 | 3.08E-43 | 87.72171 |
| VCAM1 | 0.802621 | 1.962414 | 15.21828 | 2.91E-43 | 4.75E-43 | 87.28066 |
| CXCL9 | 1.220288 | 2.591034 | 15.01103 | 2.53E-42 | 4.05E-42 | 85.1291 |
| SRGN | 0.740515 | 4.507184 | 15.01064 | 2.54E-42 | 4.05E-42 | 85.12513 |
| SAMD9 | 0.688053 | 1.366766 | 14.65976 | 9.59E-41 | 1.51E-40 | 81.50837 |
| STX11 | 0.559181 | 1.076457 | 14.5143 | 4.28E-40 | 6.69E-40 | 80.01887 |
| FCGR2A | 0.616213 | 1.843828 | 14.45719 | 7.69E-40 | 1.19E-39 | 79.43576 |
| PIM2 | 0.509933 | 2.691067 | 14.33352 | 2.73E-39 | 4.17E-39 | 78.17626 |
| IGJ | 1.581147 | 3.622943 | 14.18225 | 1.27E-38 | 1.93E-38 | 76.64187 |
| BTN3A1 | 0.564555 | 2.795558 | 14.13756 | 2.00E-38 | 3.01E-38 | 76.1899 |
| RGS1 | 0.883593 | 3.352218 | 13.94382 | 1.43E-37 | 2.12E-37 | 74.23754 |
| TGFBI | 0.658337 | 3.054374 | 13.88778 | 2.51E-37 | 3.69E-37 | 73.67503 |
| IFIT3 | 0.868699 | 2.508475 | 13.80875 | 5.56E-37 | 8.09E-37 | 72.88342 |
| VNN2 | 0.856912 | 1.414446 | 13.52823 | 9.20E-36 | 1.33E-35 | 70.09025 |
| CP | 1.199551 | 2.074071 | 13.33177 | 6.47E-35 | 9.25E-35 | 68.14983 |
| IFI44 | 0.763836 | 2.769709 | 13.04865 | 1.05E-33 | 1.49E-33 | 65.37752 |
| TAP1 | 0.592688 | 3.991737 | 12.87208 | 5.89E-33 | 8.26E-33 | 63.6633 |
| FPR1 | 0.621281 | 1.27841 | 12.55158 | 1.31E-31 | 1.81E-31 | 60.58218 |
| IRF1 | 0.668295 | 2.885772 | 12.42229 | 4.51E-31 | 6.20E-31 | 59.3506 |
| IFIT2 | 0.68498 | 2.020566 | 12.34635 | 9.30E-31 | 1.27E-30 | 58.63031 |
| RHOH | 0.43449 | 1.301234 | 12.30769 | 1.34E-30 | 1.82E-30 | 58.26459 |
| MAP3K8 | 0.551984 | 1.524416 | 12.11615 | 8.25E-30 | 1.10E-29 | 56.46163 |
| RSAD2 | 0.644918 | 1.583933 | 11.8751 | 7.91E-29 | 1.05E-28 | 54.21463 |
| EPB41L2 | 0.588997 | 2.16336 | 11.82737 | 1.23E-28 | 1.62E-28 | 53.77272 |
| MX2 | 0.524811 | 1.018473 | 11.5676 | 1.37E-27 | 1.78E-27 | 51.38515 |
| MMP2 | 0.684999 | 4.061947 | 11.52367 | 2.04E-27 | 2.64E-27 | 50.98442 |
| NUAK1 | 0.60348 | 2.057693 | 11.30533 | 1.50E-26 | 1.92E-26 | 49.00596 |
| OLFML2B | 0.586746 | 2.091495 | 11.30247 | 1.54E-26 | 1.96E-26 | 48.98017 |
| S100A9 | 0.930725 | 3.21357 | 11.15245 | 5.97E-26 | 7.53E-26 | 47.634 |
| TCF7 | 0.478345 | 1.458974 | 11.02595 | 1.86E-25 | 2.32E-25 | 46.5072 |
| SPOCK2 | 0.736903 | 2.529958 | 10.8857 | 6.48E-25 | 8.04E-25 | 45.26725 |
| ROBO1 | 0.574512 | 2.37736 | 10.11146 | 5.37E-22 | 6.61E-22 | 38.60316 |
| IL23A | 0.275268 | 1.141273 | 10.03912 | 9.91E-22 | 1.21E-21 | 37.99682 |
| IFIT1 | 0.6718 | 2.255537 | 9.862769 | 4.35E-21 | 5.26E-21 | 36.53098 |
| OLR1 | 0.548646 | 1.422743 | 9.627455 | 3.05E-20 | 3.66E-20 | 34.6024 |
| RGS16 | 0.653401 | 1.868871 | 9.376521 | 2.35E-19 | 2.80E-19 | 32.58123 |
| ATP1B1 | 0.526728 | 4.057667 | 9.051175 | 3.14E-18 | 3.71E-18 | 30.01676 |
| MAP3K1 | 0.384313 | 2.352661 | 8.64054 | 7.55E-17 | 8.85E-17 | 26.87365 |
| EGLN3 | 0.384906 | 1.51417 | 8.548042 | 1.52E-16 | 1.77E-16 | 26.18048 |
| TNFSF15 | 0.485879 | 1.889556 | 8.514814 | 1.96E-16 | 2.26E-16 | 25.93283 |
| ADORA2B | 0.390371 | 1.085889 | 8.507489 | 2.07E-16 | 2.37E-16 | 25.87834 |
| PFKFB3 | 0.443791 | 2.941044 | 8.456974 | 3.03E-16 | 3.44E-16 | 25.50347 |
| AMIGO2 | 0.508829 | 2.132335 | 8.35681 | 6.39E-16 | 7.21E-16 | 24.76512 |
| GEM | 0.576261 | 2.904895 | 8.089238 | 4.57E-15 | 5.11E-15 | 22.82552 |
| PCDH17 | 0.53976 | 1.258596 | 7.95491 | 1.20E-14 | 1.34E-14 | 21.87008 |
| DYRK3 | 0.260132 | 1.263962 | 7.936497 | 1.37E-14 | 1.51E-14 | 21.74008 |
| SOCS3 | 0.787844 | 4.095289 | 7.795068 | 3.75E-14 | 4.11E-14 | 20.74937 |
| GNG12 | 0.279612 | 4.016879 | 6.880308 | 1.79E-11 | 1.95E-11 | 14.68697 |
| KLF5 | 0.414544 | 3.425042 | 6.819834 | 2.63E-11 | 2.84E-11 | 14.30797 |
| SCUBE2 | 0.718302 | 3.371362 | 6.648501 | 7.76E-11 | 8.31E-11 | 13.24932 |
| HMGB2 | 0.259402 | 3.979275 | 6.485821 | 2.12E-10 | 2.26E-10 | 12.26505 |
| RNF19A | 0.278576 | 3.2098 | 5.828916 | 1.00E-08 | 1.06E-08 | 8.503106 |
| IL6ST | 0.273712 | 4.118024 | 5.75346 | 1.52E-08 | 1.60E-08 | 8.09328 |
| ACHE | 0.352972 | 1.814642 | 5.486737 | 6.51E-08 | 6.78E-08 | 6.682289 |
| KCNJ2 | 0.235703 | 1.285463 | 5.115398 | 4.47E-07 | 4.62E-07 | 4.817212 |
| SF3B1 | 0.157875 | 4.569933 | 4.438171 | 1.12E-05 | 1.15E-05 | 1.721682 |
| NR4A2 | 0.396231 | 2.916196 | 4.407546 | 1.28E-05 | 1.31E-05 | 1.591232 |
| SMEK1 | 0.116708 | 3.638065 | 3.933759 | 9.54E-05 | 9.67E-05 | -0.31961 |
| SEC61A2 | 0.113904 | 1.801958 | 3.832011 | 0.000143 | 0.000144 | -0.70342 |
| CALML4 | 0.090342 | 1.325501 | 2.152092 | 0.031867 | 0.031867 | -5.63852 |

TCGA: The Cancer Genome Atlas.
