# Supplementary material for: Immune response drives outcomes in prostate cancer: implications for immunotherapy
Source: Mol Oncol. 2020 Dec 29;15(5):1358–75. doi: 10.1002/1878-0261.12887 (PMC8096785; doi:10.1002/1878-0261.12887)
Supplement: Supplementary file 4 — Appendix S1. Materials and Methods. [file MOL2-15-1358-s004.docx]

**Immune Response Drives Outcomes in Prostate Cancer: Implications for Immunotherapy**

Jialin Meng^1^, Yujie Zhou^2^, Xiaofan Lu^3^, Zichen Bian^1^, Yiding Chen^1^, Jun Zhou^1^, Li Zhang^1^, Zongyao Hao^1^, Meng Zhang^1,4*^, Chaozhao Liang^1*^

1. Department of Urology, The First Affiliated Hospital of Anhui Medical University; Institute of Urology, Anhui Medical University; Anhui Province Key Laboratory of Genitourinary Diseases, Anhui Medical University, Hefei, 230022, P.R. China.
2. Division of Gastroenterology and Hepatology, Key Laboratory of Gastroenterology and Hepatology, Ministry of Health, Renji Hospital, School of Medicine, Shanghai Jiao Tong University, Shanghai Institute of Digestive Disease, Shanghai, P.R. China.
3. Research Center of Biostatistics and Computational Pharmacy, China Pharmaceutical University, Nanjing, P.R. China.
4. Urology Institute of Shenzhen University, The Third Affiliated Hospital of Shenzhen University, Shenzhen University, Shenzhen 518000, People’s Republic of China.

*Correspondence to Meng Zhang ([zhangmeng1930@126.com](mailto:zhangmeng1930@126.com)) and Chaozhao Liang ([liang_chaozhao@ahmu.edu.cn](mailto:liang_chaozhao@ahmu.edu.cn))

**Tel.:** +8655162922234, and **Fax.:** +8655162922234

**Address:** Jixi Road 218, Shushan District, Hefei City 230022, Anhui Province, People’s Republic of China.

**Supplementary Materials and Methods**

**Prostate cancer cohorts and gene expression profile**

A total of 1488 prostate cancer patients were enrolled in the current study from the public databases, with the gene expression profiles, clinicopathological features, and recurrent-free survival data. For the TCGA-PRAD cohort, we obtained the level 3 gene expression profile of 495 patients from the TCGA Data Portal (https://tcga-data.nci.nih.gov/tcga), only genes expressed in at least 50% of the samples were retained for analyses. For the further external cohorts validation, GSE116918, GSE70770, and MSKCC cohorts, the gene expression profile were collected from Gene Expression Omnibus (<http://www.ncbi.nlm.nih.gov/geo/>), which meet the data requirement for NMF, profiled by microarray (Almac Diagnostics Prostate Disease-Specific Array, Illumina HumanHT-12 V4.0 expression beadchip, Affymetrix Human Exon 1.0 ST Array). The follow-up data for all the four cohorts are all available and collected from the above-mentioned sources. Detailed information on these datasets was displayed in **supplementary table 1.**

**Real-world clinical samples collection and sequencing**

We obtained the formalin-fixed, paraffin-embedded (FFPE) tissues from 69 prostate cancer patients, who underwent radical prostatectomy in the Department of Urology, The First Affiliated Hospital of Anhui Medical University (AHMU-PC cohort). Before the FFPE sample collection, a central review of pathology was performed by an experienced pathologist. Clinicopathological characteristics were obtained from electronic records. Patients were regularly followed up by telephone, mail, or in the clinic, the endpoint of the primary outcome is the biochemical recurrence, which was defined with the presence of the PSA level greater than 0.2 ng/mL measured 6 - 13 weeks after RP, followed by a confirmatory test showing a persistent PSA greater than 0.2 ng/mL (Cookson et al., 2007). All the study designs and test procedures were performed in accordance with the Helsinki Declaration II. The Ethics approval and consent to participate in the current study were approved and consented by the ethics committee of the First Affiliated Hospital of Anhui Medical University. The detailed features of enrolled patients were described in **Table 1**. The extraction of total RNA from FFPE samples was referring to the manufacturer’s instructions provide by RNeasy FFPE Kit (Qiagen, Germany). The quality of RNA was determined by a Nanodrop (OD260/280, Thermo Fisher) and further analyzed by Agilent 2100 bioanalyzer (Agilent).

Firstly, ribosomal RNA should be removed from the total RNA (Parkhomchuk et al., 2009), and then broke into short fragments of 250-300 bp. The first strand of cDNA was synthesized by using the fragmented RNA as a template, random oligonucleotides as primers. Subsequently, the second strand of cDNA was synthesized using dNTPs (dUTP, dATP, dGTP, and dCTP) as raw materials under the DNA polymerase I system. After purification, the double-stranded cDNA was end-repaired, A-tailed, and connected with a sequencing adapter. The AMPure XP beads were employed to select the cDNA fragments of 200 bp. Then, the USER enzyme was used to degrade the second strand of U-containing cDNA, and finally, PCR amplification was performed and the library obtained. After completing the library construction, Qubit 2.0 was used to perform the preliminary quantification. And then, the library was diluted to 1.5 ng/uL, and the Agilent 2100 was used to detect the insert size of the library. After the library inspection is passed, Illumina PE150 sequencing is performed after pooling according to the effective concentration of the library and data output requirements. Bioinformatic analyses including preprocessing of raw data(Yang et al., 2013), read alignment(Garber et al., 2011; Langmead et al., 2009; Li and Durbin, 2009; Li et al., 2008), quality control(Adiconis et al., 2013; DeLuca et al., 2012), transcriptome reconstruction(Pertea et al., 2015; Robertson et al., 2010; Trapnell et al., 2010), and expression quantification(Griffith et al., 2010) were performed to obtain the final expression matrix.

**Pattern discovery of immune expression and unsupervised analysis**

In the TCGA training cohort, tumor, stromal, and immune cell transcriptome profiling data were virtually microdissected employing unsupervised NMF method as previously described (Brunet et al., 2004) via GenePattern (Reich et al., 2006). The NMF algorithm, which is suitable for decomposing biological data, can factorize the gene expression matrix V (*n* genes × *m* samples) into two matrixes: gene factor matrix*W* of (*n* genes × *k*factors) and sample factor matrix *H* of (*m* samples × *k* factors) (Lee and Seung, 1999). We chose *k* = 11 as the number of factors or expression patterns, given it could produce a high cophenetic coefficient (Brunet et al., 2004) as well as effectively decompose the dataset in our TCGA training cohort. The identification of an Immune Class, as reported similarly by Sia *et al.* (Sia et al., 2017), involved the following steps. Firstly, identification of immune-related NMF factors was achieved through a single-sample set enrichment analysis (GenePattern module “ssGSEA”) of immune enrichment score gene signature (Yoshihara et al., 2013). Among all the 11 NMF factor groups, most of the patients in the second factor were identified with the high immune enrichment score gene signature, while the average immune enrichment score of other factors is low, therefore, the second factor was then named as the “immune factor” (**Figure 2A**). We list the steps for the selection of the immune factor with GeneParttern module ‘NMF’ and ‘ssGSEA’ modules in the end after the reference. Then, the top 150 exemplar genes of the immune factor were ranked according to the descending order by the difference between factor loading value in the fifth column of matrix*W* (immune factor weight) and the largest factor loading in other columns of *W*. The function of their exemplar genes was evaluated with the enrichment by the “clusterProfiler” package (Yu et al., 2012), functional categories with an adjusted *P*-value < 0.05 were considered as significant pathways. Secondly, the top 150 exemplar genes were selected to classify into two preliminary subgroups, immune, and non-immune for the TCGA training cohort. This procedure was accomplished by supervised clustering via GenePattern module “NMFConsensus”. Finally, the immune and non-immune classes were adjusted by the multidimensional scaling (MDS) random forest method, which could visualize the level of similarity of individual cases of a dataset(Sia et al., 2017).

**Correlation of Immune Class with copy number alterations, tumor-infiltrating lymphocytes**

The tumor-infiltrating lymphocytes (TIL) abundance estimated by H&E stained whole-slide images of TCGA samples was obtained from a previous study (Saltz et al., 2018). Copy number alterations (CNA) data were generated by GISTIC2.0 from GDAC Firehose (https://gdac.broadinstitute.org). We compared the differences in amplification or deletion events of both focal and arm level between Immune and non-Immune Classes. The neoantigen number was accessed from a previous study by Rooney *et al.* (Rooney et al., 2015). The mutation data were retrieved from TCGA (https://tcga-data.nci.nih.gov); we calculated the number of nonsynonymous mutations per million bases to evaluate the tumor mutation burden (TMB). What”s more, we used the MutSigCV_v1.41(Lawrence et al., 2013) (www.broadinstitute.org) to infer signiﬁcant cancer mutated genes (q < 0.05) across the entire TCGA cohort with default parameters. Significantly differential mutations among the current three subtypes were further identified by the independent test with P < 0.05. The mutation landscape Oncoprint was drawn by R package “ComplexHeatmap” (Gu et al., 2016).

**Molecular characterization of Immune Class**

Hand-curated gene signatures representing various immune cell types or host anti-tumor immunity (Table S2) from literature and databases were used to characterize Immune Class in the TCGA cohort. Immune suppression and activation subtypes were identified by using ssGSEA (GenePattern module “ssGSEA”) and nearest template prediction (GenePattern module “NTP”) (Reich et al., 2006) of stroma activation. The signature of stroma activation was derived from Figure 2 of Moffitt et al.’s work (Moffitt et al., 2015). We explored the association between our immune molecular subgroups and four GC molecular subtypes by six pan-cancer immune subtypes (wound healing, IFN-γ dominant, inflammatory, lymphocyte depleted, immunologically quiet, and TGF-β dominant) (Thorsson et al., 2018).

Overexpression or downregulation of genes in Immune vs. non-Immune Classes was performed by “limma” package with R, genes with a false discovery rate (FDR) < 0.05, and a log_2_ fold change (FC) ≥ 1 were considered differentially expressed between two groups. Subsequently, gene set enrichment analysis (GSEA, http://www.broadinstitute.org/gsea/index.jsp) was performed to determine gene sets and pathways enriched in Immune vs. non-Immune Classes.

**Validation of immune molecular subtypes in independent external datasets**

We identified the top 150 upregulated genes between Immune and non-Immune Classes (Table S3). Then NMF-based consensus clustering based on the immune classifier was applied to identify the three immunophenotypes in three independent external datasets (Table S1) using the GenePattern module “NMFConsensus” with the150 genes. Immune-related gene signature ssGSEA scores (**Supplementary table 2**; via GenePattern module “ssGSEA”) were calculated to feature molecular characteristics and validate the existence of the abovementioned immune molecular subtypes in each dataset.

Thirty-two biopsy samples of melanoma patients receiving anti-CTLA-4 or anti-PD-1 checkpoint inhibition therapy (immunotherapy cohort) with immune-related gene profiles available (795 gene NanoString panel) were included to explore the potential of prostate cancer immune molecular subgroups for immunotherapy response prediction (Chen et al., 2016). Subclass mapping analysis (GenePattern module “SubMap”) (Hoshida et al., 2007), which reveals common subtypes in independent datasets, was applied to detect the similarity of gene expression profile between our prostate cancer immune classifier and responders of anti-CTLA-4 or anti-PD-1 in the immunotherapy cohort.

**Statistical analysis**

Comparisons of continuous data (TIL abundance, CNV, TMB, neoantigens, and signature score) between two immune molecular subtypes were performed by t-test and Wilcoxon rank-sum test for normal and non-normal distribution data, respectively. Kaplan-Meier plots and log-rank tests were employed to perform survival analysis among three immunophenotypes for recurrence-free survival. Correlations between immune molecular classification and proposed molecular subtypes were analyzed by the chi-square test. A two-sided *P* value < 0.05 was considered statistically significant. All analyses were performed by R version 3.6.5 (http://www.r-project.org).

**REFERENCES:**

Adiconis, X., Borges-Rivera, D., Satija, R., DeLuca, D.S., Busby, M.A., Berlin, A.M., Sivachenko, A., Thompson, D.A., Wysoker, A., Fennell, T., Gnirke, A., Pochet, N., Regev, A., Levin, J.Z., 2013. Comparative analysis of RNA sequencing methods for degraded or low-input samples. Nat Methods 10, 623-629.

Brunet, J.P., Tamayo, P., Golub, T.R., Mesirov, J.P., 2004. Metagenes and molecular pattern discovery using matrix factorization. Proc Natl Acad Sci U S A 101, 4164-4169.

Chen, P.L., Roh, W., Reuben, A., Cooper, Z.A., Spencer, C.N., Prieto, P.A., Miller, J.P., Bassett, R.L., Gopalakrishnan, V., Wani, K., De Macedo, M.P., Austin-Breneman, J.L., Jiang, H., Chang, Q., Reddy, S.M., Chen, W.S., Tetzlaff, M.T., Broaddus, R.J., Davies, M.A., Gershenwald, J.E., Haydu, L., Lazar, A.J., Patel, S.P., Hwu, P., Hwu, W.J., Diab, A., Glitza, I.C., Woodman, S.E., Vence, L.M., Wistuba, II, Amaria, R.N., Kwong, L.N., Prieto, V., Davis, R.E., Ma, W., Overwijk, W.W., Sharpe, A.H., Hu, J., Futreal, P.A., Blando, J., Sharma, P., Allison, J.P., Chin, L., Wargo, J.A., 2016. Analysis of Immune Signatures in Longitudinal Tumor Samples Yields Insight into Biomarkers of Response and Mechanisms of Resistance to Immune Checkpoint Blockade. Cancer discovery 6, 827-837.

Cookson, M.S., Aus, G., Burnett, A.L., Canby-Hagino, E.D., D'Amico, A.V., Dmochowski, R.R., Eton, D.T., Forman, J.D., Goldenberg, S.L., Hernandez, J., Higano, C.S., Kraus, S.R., Moul, J.W., Tangen, C., Thrasher, J.B., Thompson, I., 2007. Variation in the definition of biochemical recurrence in patients treated for localized prostate cancer: the American Urological Association Prostate Guidelines for Localized Prostate Cancer Update Panel report and recommendations for a standard in the reporting of surgical outcomes. J Urol 177, 540-545.

DeLuca, D.S., Levin, J.Z., Sivachenko, A., Fennell, T., Nazaire, M.D., Williams, C., Reich, M., Winckler, W., Getz, G., 2012. RNA-SeQC: RNA-seq metrics for quality control and process optimization. Bioinformatics 28, 1530-1532.

Garber, M., Grabherr, M.G., Guttman, M., Trapnell, C., 2011. Computational methods for transcriptome annotation and quantification using RNA-seq. Nat Methods 8, 469-477.

Griffith, M., Griffith, O.L., Mwenifumbo, J., Goya, R., Morrissy, A.S., Morin, R.D., Corbett, R., Tang, M.J., Hou, Y.C., Pugh, T.J., Robertson, G., Chittaranjan, S., Ally, A., Asano, J.K., Chan, S.Y., Li, H.I., McDonald, H., Teague, K., Zhao, Y., Zeng, T., Delaney, A., Hirst, M., Morin, G.B., Jones, S.J., Tai, I.T., Marra, M.A., 2010. Alternative expression analysis by RNA sequencing. Nat Methods 7, 843-847.

Gu, Z., Eils, R., Schlesner, M., 2016. Complex heatmaps reveal patterns and correlations in multidimensional genomic data. Bioinformatics 32, 2847-2849.

Hoshida, Y., Brunet, J.P., Tamayo, P., Golub, T.R., Mesirov, J.P., 2007. Subclass mapping: identifying common subtypes in independent disease data sets. PloS one 2, e1195.

Langmead, B., Trapnell, C., Pop, M., Salzberg, S.L., 2009. Ultrafast and memory-efficient alignment of short DNA sequences to the human genome. Genome Biol 10, R25.

Lawrence, M.S., Stojanov, P., Polak, P., Kryukov, G.V., Cibulskis, K., Sivachenko, A., Carter, S.L., Stewart, C., Mermel, C.H., Roberts, S.A., 2013. Mutational heterogeneity in cancer and the search for new cancer-associated genes. Nature 499, 214.

Lee, D.D., Seung, H.S., 1999. Learning the parts of objects by non-negative matrix factorization. Nature 401, 788-791.

Li, H., Durbin, R., 2009. Fast and accurate short read alignment with Burrows-Wheeler transform. Bioinformatics 25, 1754-1760.

Li, H., Ruan, J., Durbin, R., 2008. Mapping short DNA sequencing reads and calling variants using mapping quality scores. Genome research 18, 1851-1858.

Moffitt, R.A., Marayati, R., Flate, E.L., Volmar, K.E., Loeza, S.G., Hoadley, K.A., Rashid, N.U., Williams, L.A., Eaton, S.C., Chung, A.H., Smyla, J.K., Anderson, J.M., Kim, H.J., Bentrem, D.J., Talamonti, M.S., Iacobuzio-Donahue, C.A., Hollingsworth, M.A., Yeh, J.J., 2015. Virtual microdissection identifies distinct tumor- and stroma-specific subtypes of pancreatic ductal adenocarcinoma. Nat Genet 47, 1168-1178.

Parkhomchuk, D., Borodina, T., Amstislavskiy, V., Banaru, M., Hallen, L., Krobitsch, S., Lehrach, H., Soldatov, A., 2009. Transcriptome analysis by strand-specific sequencing of complementary DNA. Nucleic Acids Res 37, e123.

Pertea, M., Pertea, G.M., Antonescu, C.M., Chang, T.C., Mendell, J.T., Salzberg, S.L., 2015. StringTie enables improved reconstruction of a transcriptome from RNA-seq reads. Nat Biotechnol 33, 290-295.

Reich, M., Liefeld, T., Gould, J., Lerner, J., Tamayo, P., Mesirov, J.P., 2006. GenePattern 2.0. Nat Genet 38, 500-501.

Robertson, G., Schein, J., Chiu, R., Corbett, R., Field, M., Jackman, S.D., Mungall, K., Lee, S., Okada, H.M., Qian, J.Q., Griffith, M., Raymond, A., Thiessen, N., Cezard, T., Butterfield, Y.S., Newsome, R., Chan, S.K., She, R., Varhol, R., Kamoh, B., Prabhu, A.L., Tam, A., Zhao, Y., Moore, R.A., Hirst, M., Marra, M.A., Jones, S.J., Hoodless, P.A., Birol, I., 2010. De novo assembly and analysis of RNA-seq data. Nat Methods 7, 909-912.

Rooney, M.S., Shukla, S.A., Wu, C.J., Getz, G., Hacohen, N., 2015. Molecular and genetic properties of tumors associated with local immune cytolytic activity. Cell 160, 48-61.

Saltz, J., Gupta, R., Hou, L., Kurc, T., Singh, P., Nguyen, V., Samaras, D., Shroyer, K.R., Zhao, T., Batiste, R., Van Arnam, J., Cancer Genome Atlas Research, N., Shmulevich, I., Rao, A.U.K., Lazar, A.J., Sharma, A., Thorsson, V., 2018. Spatial Organization and Molecular Correlation of Tumor-Infiltrating Lymphocytes Using Deep Learning on Pathology Images. Cell Rep 23, 181-193 e187.

Sia, D., Jiao, Y., Martinez-Quetglas, I., Kuchuk, O., Villacorta-Martin, C., Castro de Moura, M., Putra, J., Camprecios, G., Bassaganyas, L., Akers, N., Losic, B., Waxman, S., Thung, S.N., Mazzaferro, V., Esteller, M., Friedman, S.L., Schwartz, M., Villanueva, A., Llovet, J.M., 2017. Identification of an Immune-specific Class of Hepatocellular Carcinoma, Based on Molecular Features. Gastroenterology 153, 812-826.

Thorsson, V., Gibbs, D.L., Brown, S.D., Wolf, D., Bortone, D.S., Ou Yang, T.H., Porta-Pardo, E., Gao, G.F., Plaisier, C.L., Eddy, J.A., Ziv, E., Culhane, A.C., Paull, E.O., Sivakumar, I.K.A., Gentles, A.J., Malhotra, R., Farshidfar, F., Colaprico, A., Parker, J.S., Mose, L.E., Vo, N.S., Liu, J., Liu, Y., Rader, J., Dhankani, V., Reynolds, S.M., Bowlby, R., Califano, A., Cherniack, A.D., Anastassiou, D., Bedognetti, D., Rao, A., Chen, K., Krasnitz, A., Hu, H., Malta, T.M., Noushmehr, H., Pedamallu, C.S., Bullman, S., Ojesina, A.I., Lamb, A., Zhou, W., Shen, H., Choueiri, T.K., Weinstein, J.N., Guinney, J., Saltz, J., Holt, R.A., Rabkin, C.E., Cancer Genome Atlas Research, N., Lazar, A.J., Serody, J.S., Demicco, E.G., Disis, M.L., Vincent, B.G., Shmulevich, L., 2018. The Immune Landscape of Cancer. Immunity.

Trapnell, C., Williams, B.A., Pertea, G., Mortazavi, A., Kwan, G., van Baren, M.J., Salzberg, S.L., Wold, B.J., Pachter, L., 2010. Transcript assembly and quantification by RNA-Seq reveals unannotated transcripts and isoform switching during cell differentiation. Nat Biotechnol 28, 511-515.

Yang, X., Liu, D., Liu, F., Wu, J., Zou, J., Xiao, X., Zhao, F., Zhu, B., 2013. HTQC: a fast quality control toolkit for Illumina sequencing data. BMC Bioinformatics 14, 33.

Yoshihara, K., Shahmoradgoli, M., Martinez, E., Vegesna, R., Kim, H., Torres-Garcia, W., Trevino, V., Shen, H., Laird, P.W., Levine, D.A., Carter, S.L., Getz, G., Stemke-Hale, K., Mills, G.B., Verhaak, R.G., 2013. Inferring tumour purity and stromal and immune cell admixture from expression data. Nat Commun 4, 2612.

Yu, G., Wang, L.G., Han, Y., He, Q.Y., 2012. clusterProfiler: an R package for comparing biological themes among gene clusters. OMICS 16, 284-287.

**We listed the steps for the selection of the most important immune factor in Figure 2A with GeneParttern module ‘NMF’ and ‘ssGSEA’ as below:**

**Step 1. Obtain the immune enrichment score of 495 patients from the TCGA-PRAD cohort**

Users should register, and open the ssGSEA module, and upload the gene matrix profile. The specific gene set of 141 immune associate genes studied in the current work were listed in **Supplementary Table 1**. Then, we could obtain the immune enrichment score for each patient to prepare for the next steps. The gene matrix file should be in GCT format, and the gene set file should be in GMT format.


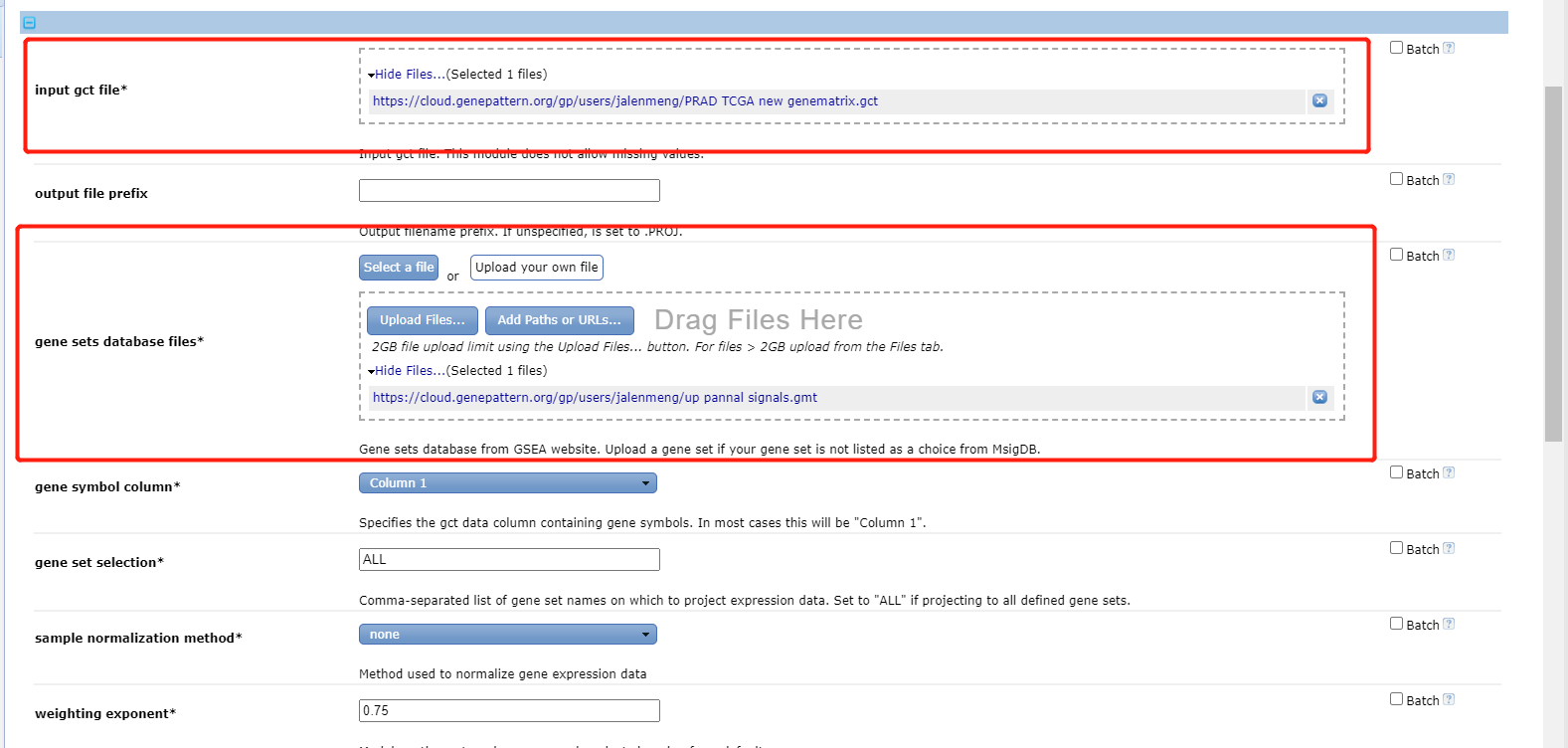


**The GCT format data need to meet the following terms.**


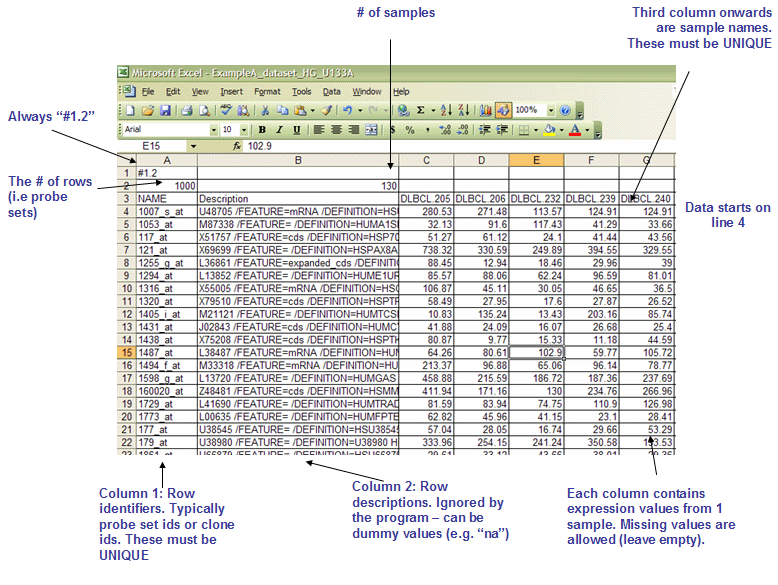


**The GCT format data need to meet the following terms.**


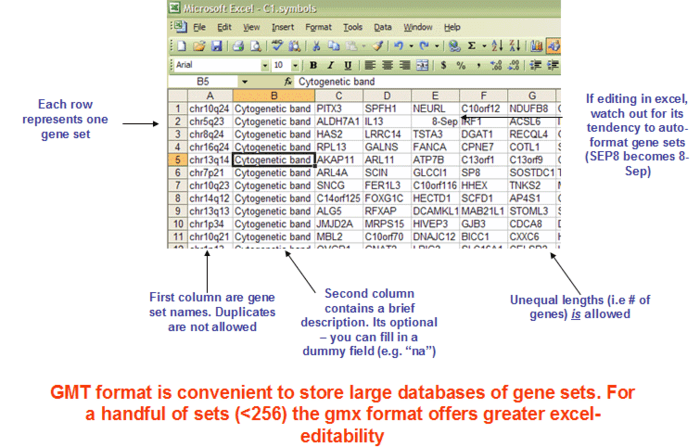


**Step 2. Prepare the format of input data for the “NMF” analysis**

The required input gene expression matrix data format is mentioned in the module, and for “NMF”, GCT format is needed.


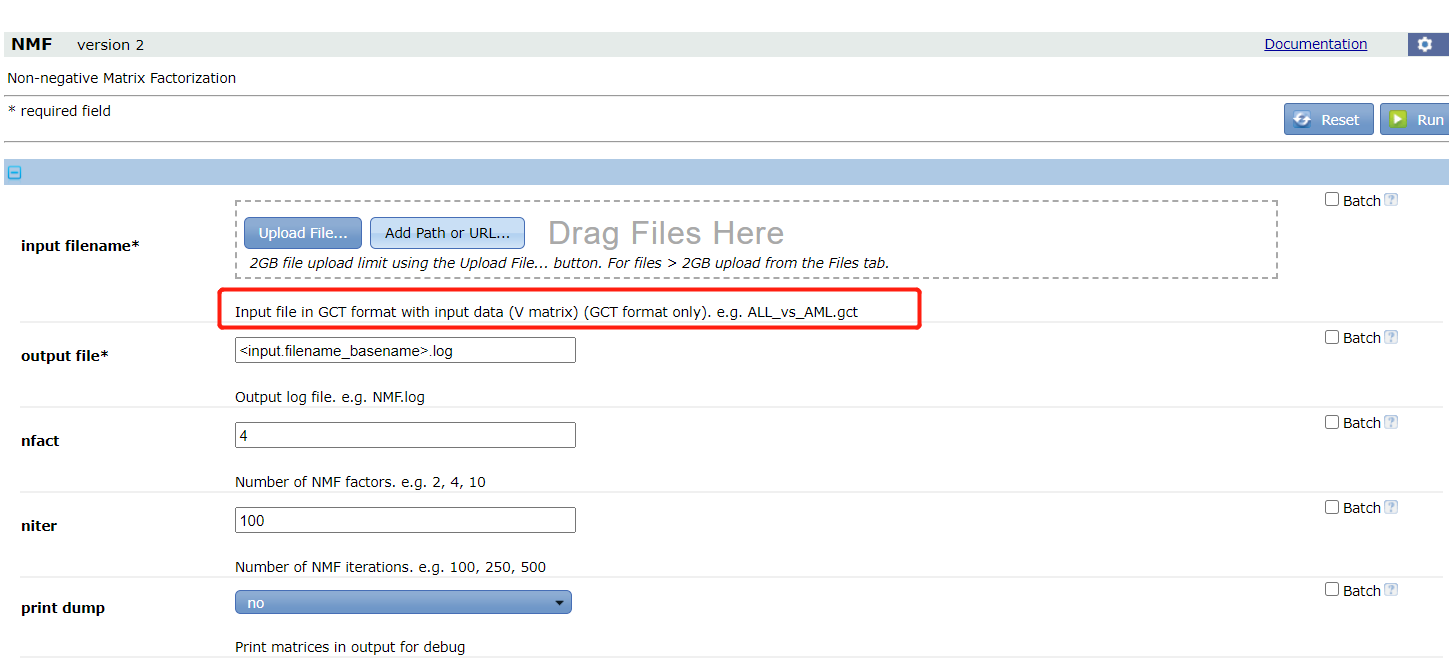


**Step 3. Upload the data into the NMF module, and set the related parameters**

The parameters need to be set as mentioned below. One of the most important parameters is ‘nfact’, in our study, we tried the number of 8,9,10,11, which could be used to separate all input patients according to different factors. It takes about 16 hours to obtain the results for each analysis.


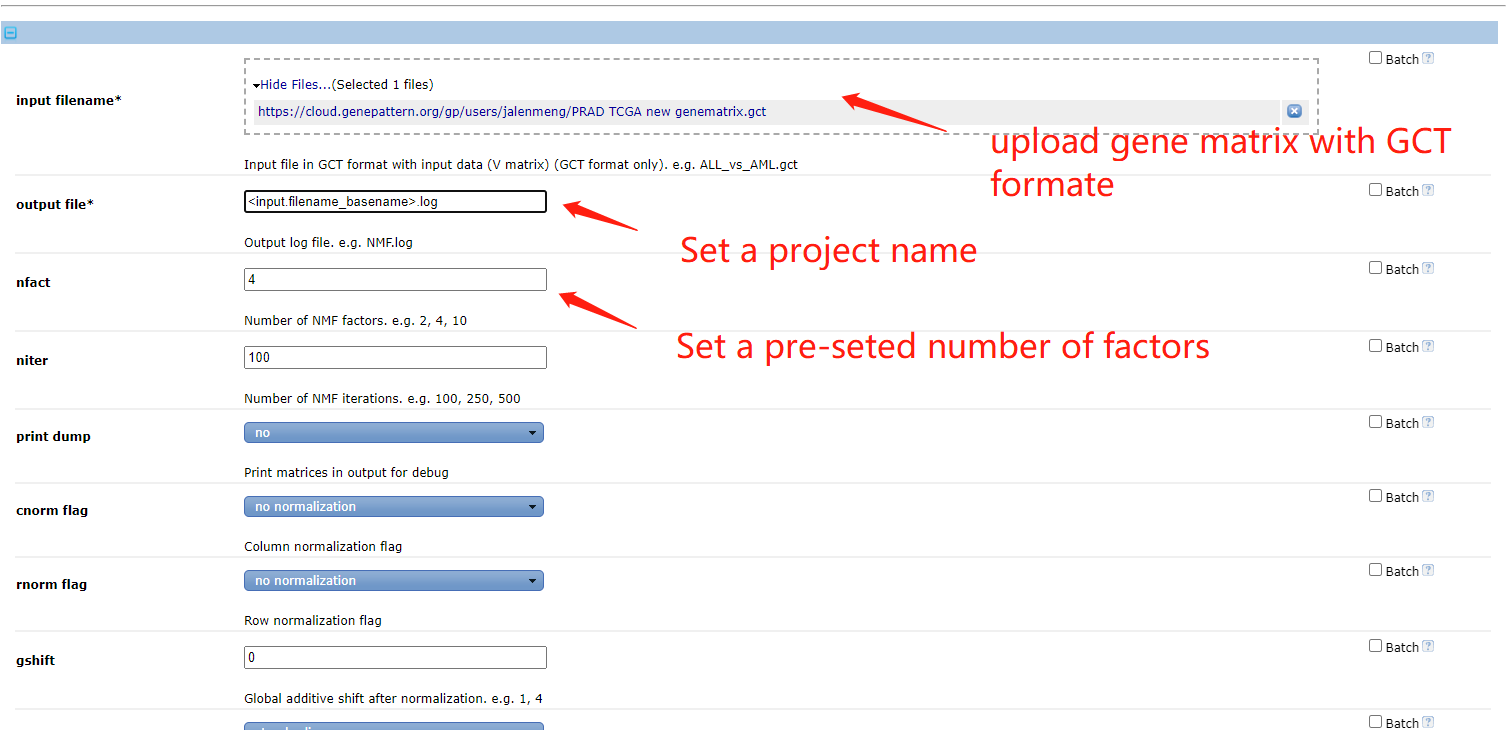


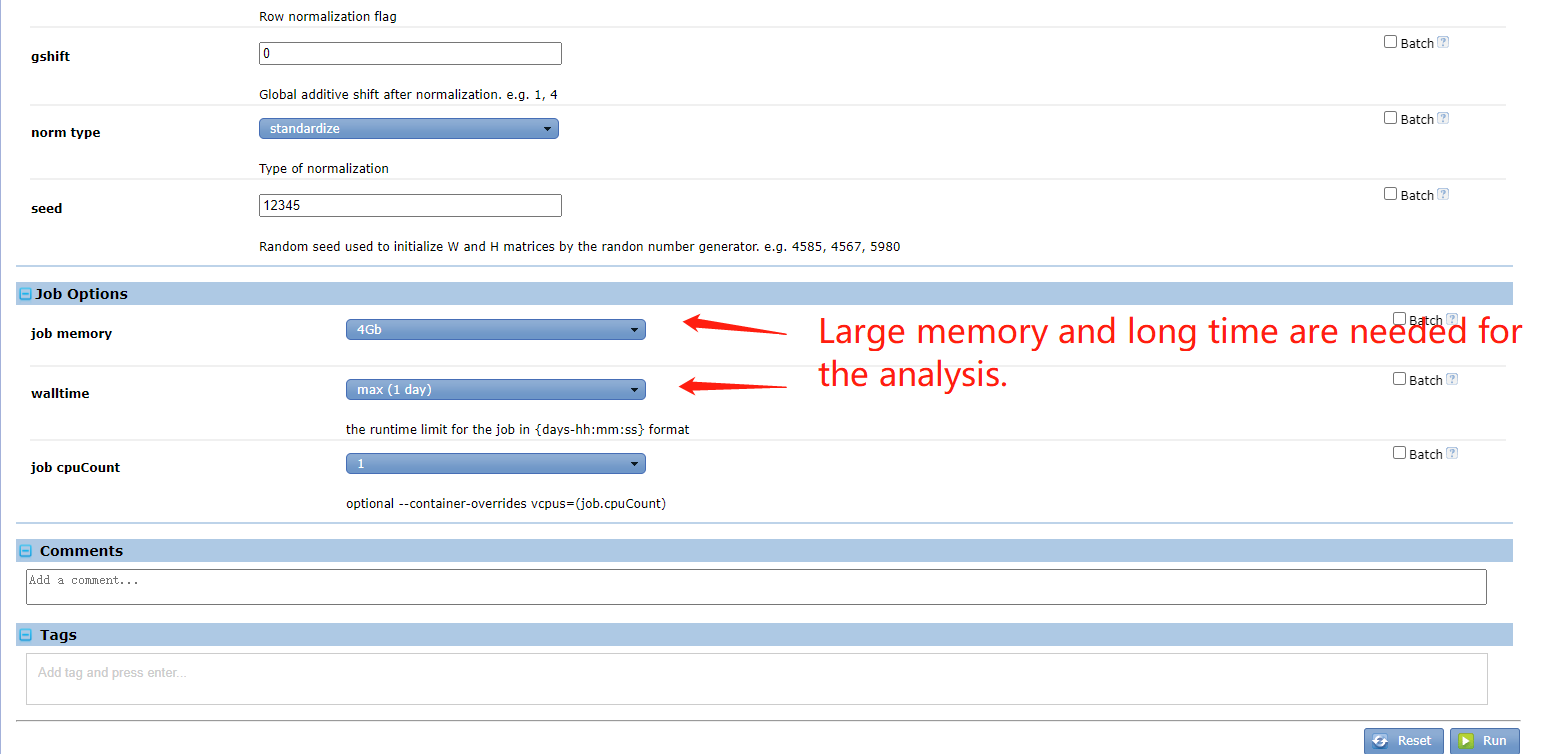


**Step 4. Select the immune factor**

After analysis, we could download the immune enrichment score for each patient from the ‘ssGSEA’ module, and download the separated factor results from the ‘NMF’ module. Then, we identified the distribution of patients with different immune enrichment scores. Here, we used ‘11 factors’, which showed the best subclassification values to obtain the ‘immune factor’. For the patients in the ‘immune factor’ subgroup, they showed the highest immune score than others.


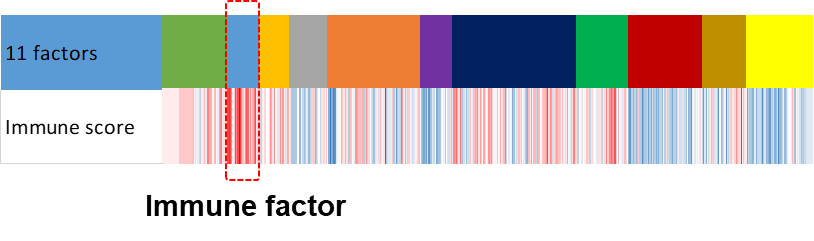


We mentioned **the most important parts above and listed in the Materials and Methods part in the main text, as well as the supplementary materials and methods.** For the other analyses, the authors could carry out according to their demands.
